# Fibrillarin evolution through the Tree of Life: Comparative genomics and microsynteny network analyses provide new insights into the evolutionary history of Fibrillarin

**DOI:** 10.1371/journal.pcbi.1008318

**Published:** 2020-10-19

**Authors:** Alejandro Pereira-Santana, Samuel David Gamboa-Tuz, Tao Zhao, M. Eric Schranz, Pablo Vinuesa, Andrea Bayona, Luis C. Rodríguez-Zapata, Enrique Castano

**Affiliations:** 1 Unidad de Bioquímica y Biología molecular de plantas, Centro de Investigación Científica de Yucatán, Mérida, Yucatán, México; 2 Unidad de Biotecnología Industrial, Centro de Investigación y Asistencia en Tecnología y Diseño del Estado de Jalisco, Zapopan, Jalisco, México; 3 Dirección de Cátedras, *Consejo Nacional de Ciencia y Tecnología*, Ciudad de México, México; 4 Unidad de Biotecnología, Centro de Investigación Científica de Yucatán, Mérida, Yucatán, México; 5 Bioinformatics and Evolutionary Genomics, VIB-UGent Center for Plant Systems Biology, Gent, Belgium; 6 Biosystematics Group, Wageningen University and Research, Wageningen, Netherlands; 7 Centro de Ciencias Genómicas, Universidad Nacional Autónoma de México (UNAM), Cuernavaca, Morelos, México; Temple University, UNITED STATES

## Abstract

Fibrillarin (FIB), a methyltransferase essential for life in the vast majority of eukaryotes, is involved in methylation of rRNA required for proper ribosome assembly, as well as methylation of histone H2A of promoter regions of rRNA genes. RNA viral progression that affects both plants and animals requires FIB proteins. Despite the importance and high conservation of fibrillarins, there little is known about the evolutionary dynamics of this small gene family. We applied a phylogenomic microsynteny-network approach to elucidate the evolutionary history of FIB proteins across the Tree of Life. We identified 1063 non-redundant FIB sequences across 1049 completely sequenced genomes from Viruses, Bacteria, Archaea, and Eukarya. FIB is a highly conserved single-copy gene through Archaea and Eukarya lineages, except for plants, which have a gene family expansion due to paleopolyploidy and tandem duplications. We found a high conservation of the FIB genomic context during plant evolution. Surprisingly, FIB in mammals duplicated after the Eutheria split (e.g., ruminants, felines, primates) from therian mammals (e.g., marsupials) to form two main groups of sequences, the FIB and FIB-like groups. The FIB-like group transposed to another genomic context and remained syntenic in all the eutherian mammals. This transposition correlates with differences in the expression patterns of FIB-like proteins and with elevated Ks values potentially due to reduced evolutionary constraints of the duplicated copy. Our results point to a unique evolutionary event in mammals, between FIB and FIB-like genes, that led to non-redundant roles of the vital processes in which this protein is involved.

## Author summary

Fibrillarins are a small group of proteins known to be essential for eukaryotic life as they are involved in several process in the cell. Changes in the amount of fibrillarins in cells can lead to incorrect translation of proteins, and is associated with various types of cancer as well as bacterial and viral responses. To date, no formal analysis of the evolution of fibrillarins through different groups of organisms such as prokaryotes, fungi, plants, and animals exists. Next-generation sequencing enhances the accessibility of great number of genome sequences from different organisms. The aim of this work is to detect the sequence stability of the fibrillarin gene copies on distant related organisms, and whether it could be associated to the changes on the organization of their genome structures. We applied a novel methodology to detect rearrangements on genomes and found evidence of evolutionary forces on plants that keep the genome region where fibrillarin is located conserved. Also, we observed that a duplication of fibrillarin occurred in mammals which led to novel or specific functions of fibrillarin duplicates.

## Introduction

Fibrillarin (FIB) is known primarily as an S-adenosylmethionine dependent methyltransferase (MTase) that catalyzes the site-specific 2'-O-methyl-ribose of ribosomal RNA (rRNA) molecules in Archaea and eukaryotes. FIB is a well-conserved protein in relation to its structure through Archaea and Eukarya [1, 2]. The typical structure of FIB consists of four main domains: the glycine/arginine-rich region (GAR domain), an intrinsically disordered region, a spacer region, the domain containing the RNA binding domain together with the MTase region, and an alpha region [1]. The 3D structure of several FIBs of Archaea and vertebrata animals (including human FIB) have been resolved by X-ray and nuclear magnetic resonance, which showed very similar central domains between archaea and human FIBs [2]. The GAR domain is not found in Archaea FIB, suggesting its later incorporation into eukaryote FIB during evolution [2]. To date, no FIB proteins have been detected in Bacteria [1, 2, 3]. In eukaryotes, FIB forms a ribonucleoprotein (RNP) complex with Nop56, Nop58, and 15.5ka proteins, and one of several C/D box small nucleolar RNAs (snoRNA); the latter guides the whole complex to the target rRNA for methylation [1, 4, 5]. Archaeal FIB proteins can form a similar RNP complex with L7Ae, Nop5, and a guide RNA to methylate pre-rRNA sequences [6]. Also, FIB can independently carry out the methylation of histone H2A in ribosomal promoters [7–9].

FIB proteins have been studied extensively in different model organisms and have gained attention in the scientific community due to their essential roles in cell survival, cancer therapy, stress tolerance, and nucleolar dynamics [1, 10, 11]. An early experiment in yeast identified impaired ribosomal processing, including impaired rRNA methylation, in thermos-sensitive FIB-deficient mutants [12]. Recently, a novel ribonuclease function was described for FIB; this activity is dependent on the GAR domain and is impaired by phospholipids [13]. Specific localization in the nucleoli and Cajal bodies of cells has made it possible to use it as a nucleolus marker. Fibrillarin can act as a sensor of cellular stress and change its localization to the cytoplasm [14]. Due to its role in aiding rRNA processing of ribosomal particles, fibrillarin has shown potential as a therapeutic target for some types of cancer, such as breast cancer [15]. Also, several viruses hijack FIB for the viral progression of plants and mammals [16]. In plants, FIB is involved as part of the Pol II transcription complex along with Med19a and the non-coding RNA ELF18-INDUCED LONG NONCODING RNA 1. In this context, FIB functions as a negative transcriptional regulator of immune responsive genes, including PR1 [17].

Despite the vital role of FIB for organisms and extensive molecular and biochemical studies on their functions [12, 18, 19], there are no comprehensive studies on its evolutionary history across the Tree of Life. No complete evolutionary history across the Tree of Life can exclude the viral world [20]. Viruses transfer genetic material to living organisms and therefore have a high impact on biodiversity [21]; certain lineages of viruses present a strong correlation with specific lineages of the Tree of Llife (e.g., Archaea are host of dsDNA/ssDNA viruses but not RNA viruses while ssRNA(+) viruses are widespread across Eukarya [20]). Giant viruses that can harbor up to 2500 protein coding sequences, present an expanded metabolic diversity (e.g., components of glycolysis, gluconeogenesis, TCA cycle, among other metabolic pathways), and present genome sizes up to 2.5 Mb [21–25]. A comparative genomic study of FIB protein across multiple lineages would facilitate our understanding of its molecular evolution and current functional dynamics. The reconstruction of evolutionary history requires the selection and comparison of a set of genomes to each other and outgroup species, homology-based protein detection among different lineages, the connection between compared genes and their biological function, and the genomic context, both ancient and recent rearrangements [26]. Thanks to the rapid increase in the number of fully sequenced genomes, more resolution of genomic evolutionary processes are known at smaller genome scales (e.g., gene loss and gene duplication) and entire genome-scale (ancient whole-genome duplication and triplication, WGD/WGT, or paleopolyploidy). These duplication events create genetic novelty and provide the raw material for evolution and biological diversity [27–31].

In comparative genomics, synteny analysis has proven to be a powerful tool for understanding genome rearrangements on both small and entire genome scales. Syntenic genes referred to as syntelogs, onhologs, or syntenic homologous genes, are genes that share the same genomic context. A phylogenomic and microsynteny analysis on 60 plant genomes generated clues about how evolutionary processes (genome diversification) leads to differential biochemical properties, distinctive gene expression patterns, and specific-gene expansion [32], shedding light on the contribution of evolutionary mechanisms to plant adaptations in restrictive environments. Phylogeny and a microsynteny network approach on 171 fully sequenced genomes from the Tree of Life unravelled the evolutionary history of TMBIM protein family. Synteny revealed that some groups of genes from monocots transposed to another genomic context during evolution; specific patterns of gene duplication in angiosperms were also observed [33]. Recently, [34] an in-depth network-based phylogenomic synteny analysis on 87 mammalians and 107 angiosperm genomes identified long-term conservation and several lineage-specific patterns of evolution related to the genomic context of genes. Interestingly, several “rebel-genes” that transpose to another genomic context in mammalian genomes and conserved single-copy genes in angiosperms were detected.

We provide a phylogenetic overview of FIB proteins across the Tree of Life by using 1049 available complete sequenced genomes spanning viruses, Bacteria, Archaea, protists, fungi, plants, and animals. Additionally, we applied a novel microsynteny network approach on fungi, plants, and animals to unravel the evolution of genome structure and its consequences on FIB dynamics. Our goal is to identify patterns of genome evolution to provide insights into how genome dynamics may have contributed to trait evolution. Our evolutionary analyses provide the first comprehensive survey of FIB proteins over four billion years of evolutionary history.

## Results

### The fibrillarin family through the tree of life: General trends

We performed a homology search for FIB proteins across 1049 completely genome-sequenced organisms from the three domains of life (Bacteria, Archaea, and Eukarya) and viruses (For discussion about the fourth domain please see [35]). We sought to identify FIB-like proteins in 47 proteomes of viruses that: infect Bacteria and Archaea; derive from ancient symbiotic viruses; and belong to several species of giant viruses from different lineages such as Pandoravirus, Pithoviridae, Megaviricetes (Phycodnaviridae, Mimiviridae, and Marseillevirus), Faustovirus, Pacmanvirus; as well as some giant uncultured marine viruses from environmental samples (S1 Table). No significant match against viruses was detected. Similarly, no FIB sequences were detected in any of the 212 bacterial genomes analyzed (182 well described bacteria [WDB] [36] and 30 species from the candidate phyla radiation [CPR] group [36, 37]; S2 Table), consistent with previous reports [2, 3]. Among CPR bacteria, we detected 20 different families from the MTase superfamily, but none were significant against our HMM-FIB model for FIB proteins (S3 Table). Searching for the 15.5k protein (L7Ae homolog in Archaea) and the NOP56 protein (NOP5 homolog in Archaea) across Bacteria proteomes did not result in any significant hit against this lineage (S4 Table). These proteins are essential for the recruitment of fibrillarin in the RNP complex.

**Fig 1:**
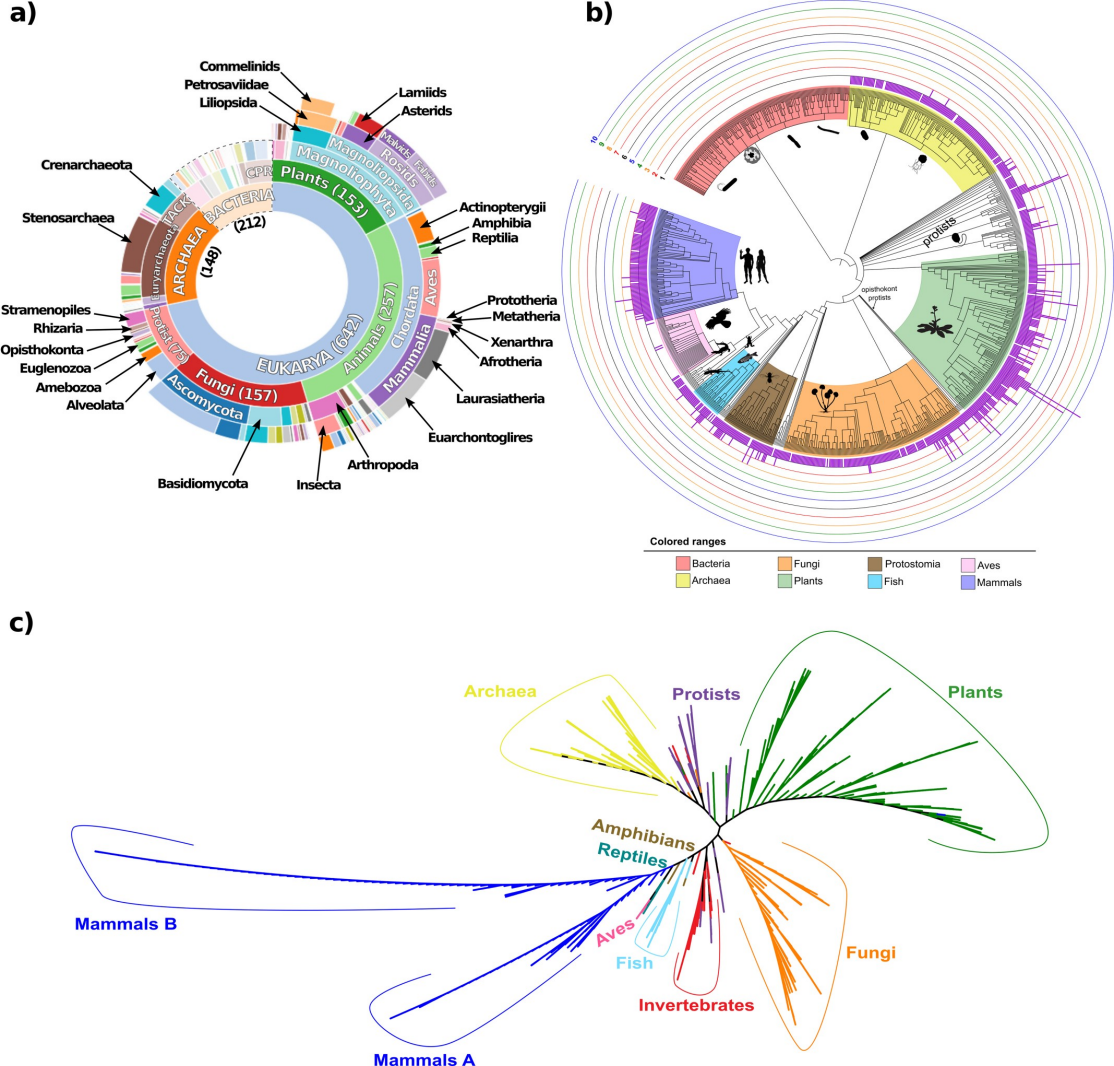
Overview of inspected taxonomic groups and distribution of FIB protein sequences through the three domains of life. a) Depiction of the major taxonomic groups analyzed in this study. FIB sequences were sought across a total of 1002 genomes from the three domains of life (212 Bacteria, 148 Archaea, and 642 Eukarya). Graph dimensions are not to scale. b) The number of FIB sequences (purple bars) per analyzed genome are grouped according to major taxa in a species phylogenetic tree. Concentric circles indicate the number of FIB sequences. c) Unrooted phylogenetic tree of the total 1063 FIB proteins found in Archaea and Eukarya, colored by main taxonomic groups.

We identified 1063 non-redundant FIB sequences spanning all major clades from Archaea and Eukarya (S5–S9 Tables). The analyzed homologous sequences include: 143 in Archaea (from 148 analyzed genomes), 103 in protists (from 75 genomes), 170 in fungi (from 157 genomes), 328 in plants (from 153 genomes), and 319 in animals (from 257 genomes; Fig 1A and 1B). We then inferred the phylogenetic gene tree from the alignment of the 1063 retrieved sequences (Fig 1C; S1 Fig; S10 Table). Based on the tree, almost all analyzed sequences formed monophyletic groups according to their taxonomic affiliation. Notably, mammalian FIBs formed into two separate monophyletic groups (Mammals A and B).

In order to identify amino acid conservation of the FIB domain across lineages, (S2 Fig) was built an alignment by Clustal-O with default parameters using representative sequences from different lineages chosen to encompass different phyla and classes across the five major analyzed groups (e.g., for animals we used representative sequences of porifera, cnidaria, mollusca, artropoda, tardigrada, nematoda, fish, coelacanth, amphibia, reptiles, and mammals). Overall, FIB domain composition is well conserved from Archaea to lineages of Eukarya, specifically on the MTase domain (S2 Fig). FIB proteins from archaeal organisms are shorter than those from most eukaryotic organisms, but several well conserved amino acids were identified even in distant eukaryotic lineages (red shaded columns in S2 Fig). The GAR domain was not present in Archaea FIB proteins, suggesting acquisition during early eukaryotic evolution. Three different Hidden Markov Models (HMM) for RG-rich regions reported in [13] were used identify whether other RG-rich regions (like the GAR domain in FIB) were present in other proteins from Bacteria and Archaea, we had examined; no significant match was found against the proteomes of prokaryotes (S4 Table).

In Archaea, we detected FIB homologs in 140 of 148 species (95%), showing evidence of independent gene losses in eight organisms (5% S5 Table) during evolution, or due to genome annotation errors or missing data. Genomes from three Euryarchaeota species contained FIB duplicates: *Archaeoglobales archaeon* ex4484_92, *Aciduliprofundum boonei* T469, and *Halonotius* sp. J07HN4 (S5 Table). Significant hits were found in the vast majority of the 148 analyzed proteomes from Archaea for the 15.5k protein (142 significant hits) and the NOP56 protein (145 significant hits); at least one well conserved copy per specie was identified for each protein (S4 Table).

**Fig 2:**
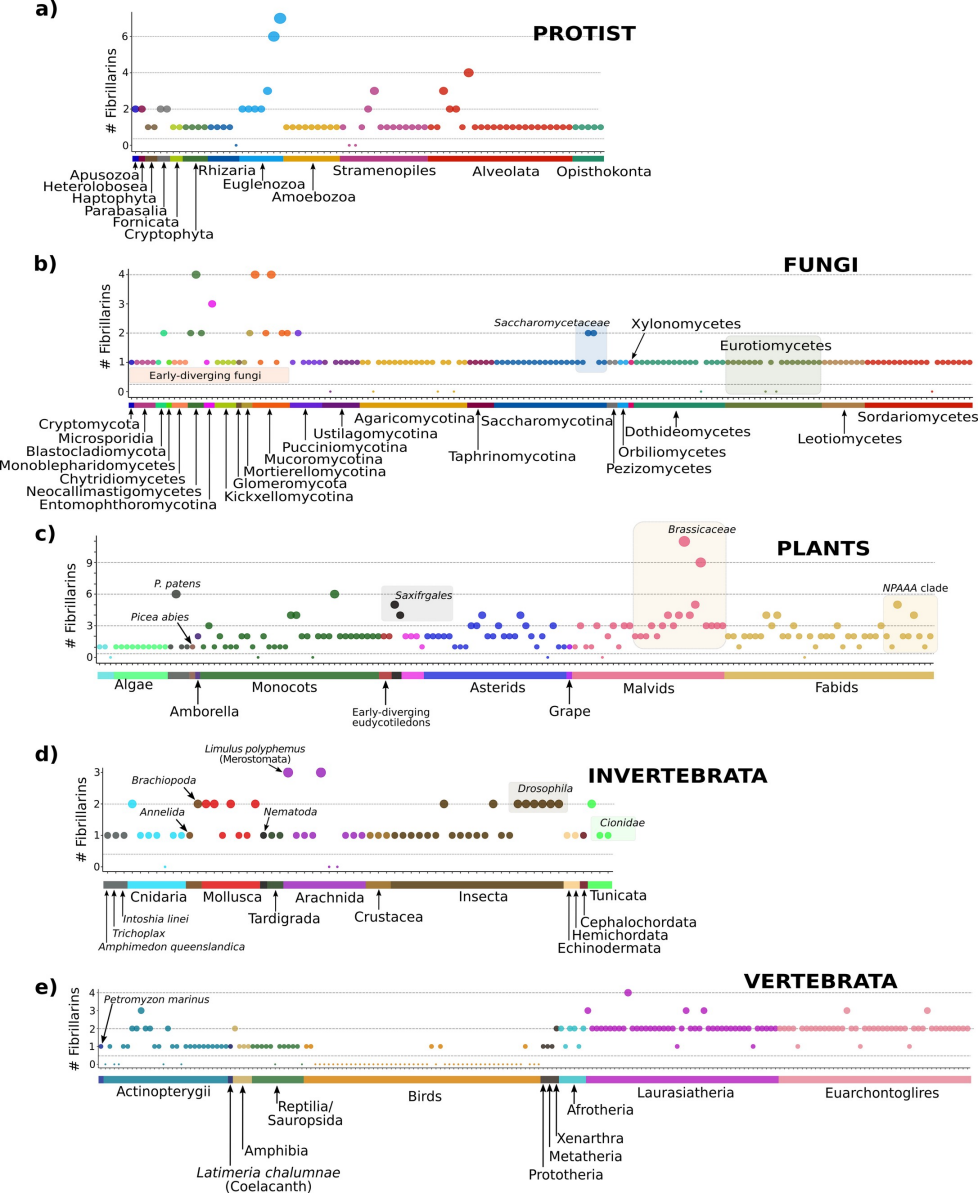
Depiction of the number of FIB proteins detected in Eukarya. a) FIB proteins detected in the protist group. b) FIB proteins detected in Fungi. c) FIBs detected in Plant genomes. d) FIBs detected in invertebrates. e) FIBs detected in vertebrates. Each circle represents the number of FIBs per species (y-axis). Each taxonomic group is presented in a unique color (dots and strips under the x-axis) consistently throughout the text and figures.

In protists, we detected 103 FIB homologs in 73 of 75 (96%) analyzed species (S6 Table). Fifty-three of 75 species (73%) contained only one FIB sequence per genome (Fig 2A). Sixteen of 75 species (23%) contained FIB duplicates ranging from 2 to 7 paralogs per genome (S6 Table). Species from Stramenopiles and Alveolata also contained more than one FIB per genome (Fig 2A). The phylogenetic analysis of protist FIBs was congruent with the major phylogenetic groups; however, the Alveolata FIBs were separated into several clades, mainly Apicomplexa and Oligohymenophorea, indicating higher divergence (S3 Fig). Interestingly, the FIB sequence SymiT_OLP95976.1 from the ancestral dinoflagellate *Symbiodinium microadriaticum* (clustered within the Apicomplexa clade, sister to dinoflagellates) contained up to 53 exons (S3 Fig).

In Fungi, we searched 157 genomes from eight major phyla [38, 39] and found a total of 170 FIB proteins in 149 species (95%; S4 Fig. and S7 Table). We did not detect FIB sequences in eight of the 157 species (5%) which were distributed through Basidiomycota and Ascomycota phyla, indicating possible independent losses during Dikarya evolution, genome annotation errors or missing data (Fig 2B; S4 Fig). Fourteen of 157 species (9%) contained FIB duplicates ranging from 2 to 4 paralogs per genome (S4 Fig). Eleven of these 14 species occur in the early-diverging groups of fungi (Fig 2B). *Zyhosaccharomyces bailii* and *Kazachstania africana* from the Saccharomycetaceae family, and *Melampsora larici-populina* from Pucciniomycotina were the only three species within the Dikarya subkingdom that contained FIB duplicates, with two paralogs each (S4 Fig). The number of exons in the fungal FIB mRNAs ranged from one to thirteen, with an average of 3.5 exons per sequence. Interestingly, in the Saccharomycotina subphylum, 18 out of 21 (86%) FIBs were intronless (S5 Fig).

In plants (*sensu lato*; 140 plants and 13 algae), we detected 328 FIBs in 147 of 153 (96%) analyzed genomes (S8 Table). For the 6 species where we could not find FIBs proteins this was likely due to genome annotation errors due to large plant genome sizes and the short-read sequencing technologies implemented for their assembly (short read technologies cannot accurately assemble large repetitive and low complexity regions). Twelve of the 13 algae (10 Chlorophyta and 2 Rhodophyta) contained only one FIB protein, the exception was *Porphyra umbilicalis* in which no FIB protein was detected (Fig 2C). For most first branching land plant lineages (Embryophyta and Tracheophyta [*Sphagnum fallax*, *Marchantia polymorpha*, and *Selaginella moellendorffii]*), we detected one FIB. The exception was *Physcomitrella patens* with 6 FIB proteins, likely due to the two rounds of ancient WGD in this lineage [40]. In *Picea abies* (Pinophyta), the only gymnosperm analyzed, we detected one FIB sequence.

All angiosperms share an ancient WGT. From the basal angiosperm *Amborella trichopoda*, we detected two FIB sequences. Monocots had FIBs that ranged from 1 to 4 paralogs per genome with an average of 1.8 FIBs per genome (BOP clade [grasses from Poaceae family] contained exactly two sequences), except for the allohexaploid *Triticum aestivum* (common wheat) that contained 6 paralogs. The early-diverging eudicot plants *Nelumbo nucifera* and *Aquilegia coerulea* contained two FIB proteins each, as did the members of the Caryophyllales clade (except for *Spinacia oleracea* with one FIB). Asterids had a range of FIBs from 1 to 4 paralogs (averaging 2 FIBs per genome). Grapevine (*Vitis vinifera*) seems to have retained only one copy of the FIB gene after the WGT event (At-γ). In the Fabids (Eurosids I), the number of FIBs ranged from 1 to 5 (with an average of 2.1 FIBs per genome); In the Malvids (Eurosids II), the FIBs ranged from 1 to 11 paralogs (an average of 3.1 FIBs per genome). The Brassicaceae family showed a dynamic increase in the number of paralogs from 2 to 11, derived from two rounds of ancient WGD (At-α and At-β) and specific WGT (Br-α) in Brassica species.

In animals (Metazoa), we analyzed 257 genomes from which we were able to detect 319 FIB sequences. We classified this group in two principal subgroups: Invertebrata, and Vertebrata. From Invertebrata, we detected 78 FIB sequences in 59 of 62 (95%) analyzed genomes (S9 Table); almost all species contained one FIB sequence. The exceptions were some members of the Mollusca clade that contained two FIBs, including *Lingula anatina* from Brachiopoda phylum (Fig 2D). The other notable group was the Diptera clade, especially the Drosophilidae family, which had two FIBs in each species.

In Vertebrata, we detected 241 FIB sequences in 140 of 195 inspected genomes (72%). We obtained a single FIB sequence in each of the species from the ancient vertebrate lineage of the lamprey (*Petromyzon marinus*), passing through actinopterygii, to the "living fossil" the Coelacanth *Latimeria chalumnae*; the Salmonidae family was the exception to this count. Since this clade contains an extra WGD event called 4R, the number of FIB sequences ranged from 2 to 3 (Fig 2E).

Amphibia and sauropsida clade also contained one FIB gene per genome, except for the allotetraploid frog *Xenopus leavis* with two FIB sequences. The Bird clade was notable because we only partial FIB sequences were detected, in only 5 of 53 (9%) inspected genomes. Two of these 5 avian species belong to the palaeognathae clade (*Struthio camelus australis* and *Tinamus guttatus*), and the other three belong to the neognathae clade (*Falco peregrinus*, *Haliaeetus leucocephalus*, and *Geospiza fortis*). Representative mammal species from Prototheria (1 species [*Ornithorhynchus anatinus*]), Metatheria (2 species [*Sarcophilus harrisii* and *Monodelphis domestica*]), Xenarthra (1 species [*Dasypus novemcinctus*]), Afrotheria (6 species [e.g., clade including Sirenia, Elephantidae, Macroscelididae, among others]), Laurasiatheria (43 species [e.g., clade including Chiroptera, Felidae, Cetacea, Ruminantia, among others]), and Euarchontoglires (43 species [e.g., clade including Dermoptera, Rodentia, Primates, among others]), were analyzed proportionally to the number of well-sequenced organisms per clade. Prototheria and Metatheria species contain only one FIB protein each, but the Xenarthra species *D*. *novemcinctus* (basal Eutheria species) has two FIB sequences. The Afrotheria, Laurasiatheria, and Euarchontoglires maintain a very marked pattern of two FIB Proteins each (Fig 2E).

### Phylogenomic microsynteny-network approach to elucidate the evolutionary history of FIB proteins in higher eukaryotes

**Fig 3:**
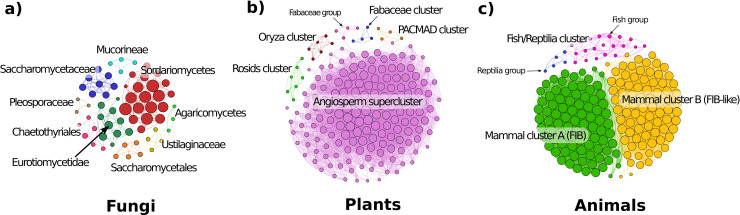
Microsynteny networks of FIB genes of the three major Eukaryotic taxa. a) Microsynteny networks of FIB genes in Fungi. Nine microsynteny communities from three different phyla (Mucoromycota, Basidiomycota, and Ascomycota). b) Microsynteny networks of FIB genes in plants formed six synteny clusters: a synteny supercluster for all angiosperms (purple nodes), and five small synteny clusters for specific clade such as Rosids (green nodes), Fabaceae (blue nodes), PACMAD (orange nodes), Oryza-specific cluster (red nodes), and a small Fabaceae group (pink nodes) that poorly linked to the Angiosperm supercluster (one link). c) Microsynteny networks of FIB genes in animals. Three major clusters include a specific Fish-Reptilia syntenic cluster (pink and blue nodes), and two mammalian-specific syntenic clusters (green and yellow nodes). Nodes represent FIB genes, and edges represent synteny relationships between them. Nodes sizes are proportional to the number of synteny connections they share. All depicted microsynteny networks were clustered by Clique percolation method (k-clique = 3) to find densely connected communities.

For all detected orthologous and paralogous FIBs sequences, we identified all syntenic FIB genes by pairwise inter- and intra-species microsynteny block detection. To decipher the evolutionary history of FIB genes in major eukaryotic lineages, we implemented a microsynteny-network approach coupled to phylogenetic profiling (“phylogenomic synteny profiling” as described by [34]) on fungi, plants, and animals. In these networks, nodes represent genes and edges represent a syntenic relationship between them. As reported by [34], some clades (such as primates and Brassicaceae) were overrepresented due to research sampling biases. Fig 3 depicts the general topology of the clustered microsynteny networks for fungi, plants, and animals.

Fungi contain nine syntenic communities, one belonging to the early-branching fungi Mucoromycotina, two from Basidiomycota, and six from Ascomycota (Fig 3A). In plants, we detected five syntenic communities: a major supercluster contained almost all detected syntenic FIB from all angiosperms (including the basal Magnoliophyta *Amborella*); another minor cluster was specific to Rosids; an *Oryza*-specific community (containing one representative for each of the six analyzed rice species); a Fabaceae-specific community; a grass PACMAD community; and a small Fabaceae group that was poorly linked to the Angiosperm supercluster (Fig 3B).

In animals, only the Vertebrata subgroup presents syntenic connections between species. We found three significant communities, one for Fish and Reptilia (containing the Coelacanth *L*. *chalumnae*), and the other two for mammals (Fig 3C). Interestingly, in mammals, one syntenic community was specific for FIB genes (Mammal cluster A), and the other was specific for the entire set of FIB-like genes (Mammal cluster B). Therefore, suggests that FIB and FIB-like genes are in different genomic contexts before the split of the Eutheria clade.

### Phylogenomic microsynteny network analysis in Fungi

**Fig 4:**
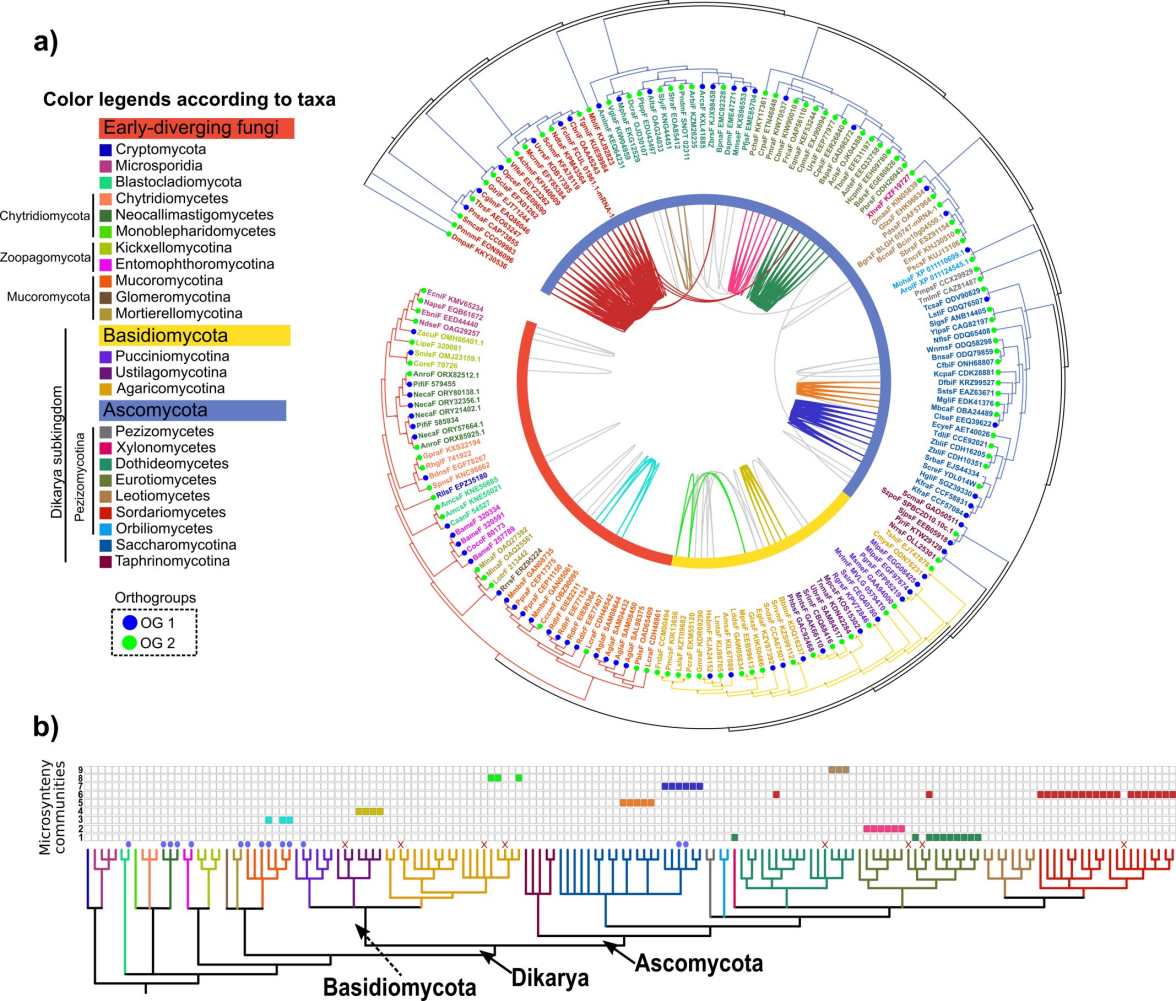
Phylogenomic microsynteny analysis of the fungal FIB homologues. a) Phylogeny of the 170 FIB proteins identified in fungi. Tree leaves are labelled by color according to main taxonomic groups, as indicated in the legend (left). The color of inner strips is by major groups: early-diverging fungi (red), Ascomycota (blue), and Basidiomycota (yellow). Internal pairwise connections between tree leaves represent pairwise synteny relationships and are colored to indicate the nine fungal microsynteny communities; gray connections represent synteny pairwise relationships not included in any community. b) Phylogenetic profiling of the microsynteny communities of FIB proteins found in fungi. The cladogram at the bottom represents analyzed fungal species; branches are colored by main taxonomic groups, as indicated in the left legend. The presence or absence of the synteny communities in each species shown in the matrix above the cladogram. Closed figures indicate the presence of a microsynteny community.

Phylogenetic analysis divided the fungal fibrillarins according to the major taxonomic groups (Fig 4A and S5 Fig). Orthology analysis (no synteny evidence) revealed the presence of two orthologous groups (OG, orthogroups) of fungal fibrillarin proteins (Fig 4A). Only the SrbaF_EJS44334 sequence from *Saccharomyces arboricola* did not belong to any OG. General phylogenomic profiling (Fig 4B) shows the affiliation of the microsynteny communities according to the fungal species tree according to colored squares.

The FIB microsynteny network of fungi comprised 97 nodes representing 57% of the total 170 fungal fibrillarin proteins identified and 239 edges (pairwise syntenic relationships; S11 Table). Clustering analysis revealed 9 syntenic communities of FIB proteins in fungi, termed F-1 to F-9 (S6 and S7 Figs and S12 Table). The number of nodes in individual communities ranged from 3 in F-8 and F-9 to a 21 in F-6. Each fungal syntenic community was mainly composed of FIB proteins from closely-related species within families, orders, or classes. The F-1 and F-6 communities shared a node from *Aspergillus aculeatus*, connecting the Eurotiomycetidae family and the Sordariomycetes class. In addition, the F-1 community contained a protein from *Xylona heveae*, and the F-6 community contained a protein from *Baudoinia panamericana*, connecting such communities to the Xylonomycetes and Dothideomycetes classes, respectively (arrows in S6 and S7 Figs).

In *Rhizopus delemar*, an ancient whole-genome duplication was detected [41], correlating with the presence of four FIB paralogs (two of which are syntelogs; S6 Fig, F-3 community). We did not find the FIB identifier among the onhologs listed by [41]. Other early-diverging fungi such as those within the Neocallimastigomycetes class contained several FIB duplications. Within Saccharomycotina, we found two syntenic communities of FIB syntelogs corresponding to the CTG-Ser clade (F-5) and the Saccharomycetaceae family (F-7). The CTG-Ser clade is composed of the Metschnikowiaceae and the Debaryomycetaceae families, which translate the CTG codon to Serine instead of Leucine [42]. The FIB homologue of *Babjeviella inositovora*, the most basal species from the CTG-Ser clade [43], was the only FIB homolog not included within the F-5 community (S6 Fig).

The FIB homolog from *Xylona heveae* retains synteny with *Ucinocarpus reesii* and *Coccidioides posadasii*; the FIB protein from *Baudoinia panamericana* retains synteny with *Chaetomium globosum* and *Thielavia terrestris;* finally, the FIB sequence from *Aspergillus aculeatus* retains synteny with five and three species from Eurotiomycetes and Sordariomycetes, respectively (S6 and S7 Figs). The result suggests that the current genomic contexts of these FIB proteins had a common origin in the last common ancestor of Pezizomycotina. Therefore, they changed by extensive and rapid lineage-specific genomic rearrangements (S4 Fig).

To examine the genomic context of each community, we retrieved all syntelog pairs contained within the same syntenic block as the FIBs homologs (S11 Table indicates the block indexes in the first column; see also S13 Table). Then annotations were assigned to all proteins/nodes from each network and low containing sub-clusters (k-clique = 3) were filtered out; the remaining networks are displayed (S8 Fig). A summary of the best-annotated proteins within the same syntenic blocks as fungal FIBs is in S14 Table.

### Phylogenomic microsynteny network analysis in plants

**Fig 5:**
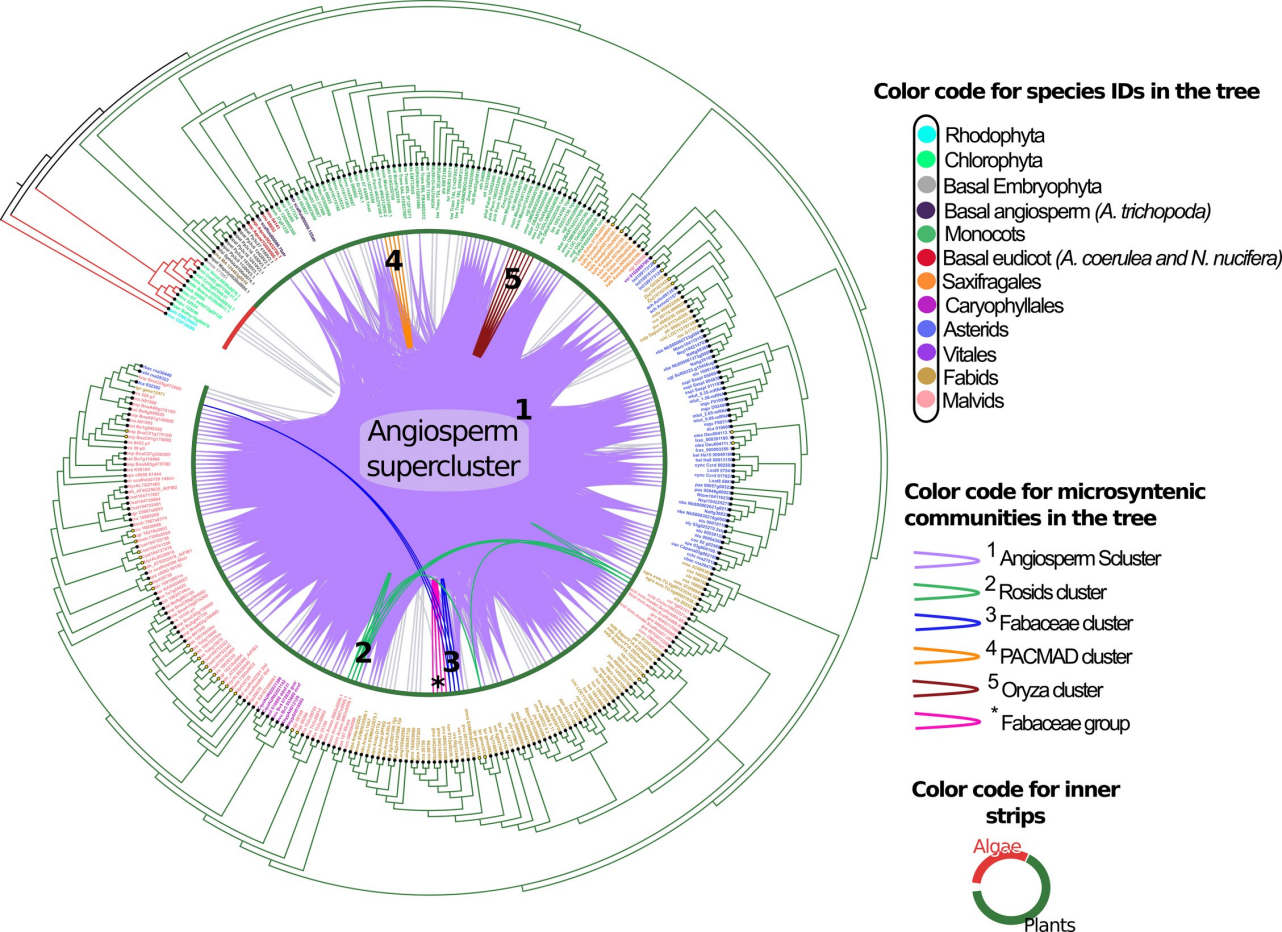
Phylogenomic microsynteny analysis of plant FIB homologues. Phylogeny of the total 327 FIB proteins detected from 153 plant genomes (13 algae and 140 plants). Names of genes are placed on the tree by taxonomic affiliation, as indicated on the right. Colors of inner strips are according to major taxonomic groups: algae (red) and angiosperms (green). Internal pairwise connections between tree leaves represent pairwise synteny relationships and are colored according to the detected microsynteny clusters, as shown in Fig 2B and S9 Fig. Gray connections represent synteny pairwise relationships not included in any community. Black filled circles on the tip of the leaves represent genes belonging to the only orthogroup detected in plants. Yellow filled circles represent tandem duplicated genes and part of the unique orthogroup.

Phylogenetic analysis shows that plant FIB proteins are monophyletic. The algae FIB proteins were all clustered together in the base of the tree, following the basal Embryophyta species (mosses, liverwort, and hornwort), then the gymnosperm *P*. *abies*, *Amborella*, the monocot species, and finally the Eudicotyledons (Fig 5). All plant FIB proteins belong to a unique orthologous group (OG; black filled circles on the tip of the leaves).

The plant FIB microsynteny network comprised 223 nodes or genes (67.9% of the 328 total plant FIB proteins identified) and 6016 edges (pairwise syntenic relationships; S15 Table). Clustering analysis revealed five microsyntenic communities of FIB proteins in plants (Fig 5; S16 Table). These are the Angiosperm supercluster (community #1 with 168 genes; purple cluster), the Rosid cluster (community #2 with 8 genes; green cluster), the Fabaceae cluster (community #3 with 4 genes; blue cluster), the PACMAD cluster (community #4 with 4 genes; orange cluster), and an *Oryza* cluster (community #5 with 6 genes; dark red cluster). From this analysis, it appears that plant FIBs remain conserved in the same ancestral syntenic block (Angiosperm supercluster). However, four syntenic communities (two for monocots [clusters 4 and 5] and two for eudicots [clusters 2 and 3]) are transposed to another genomic context (a genomic block that moved to the different genomic region and shared for those species in question). The Angiosperm microsynteny supercluster has FIBs from the basal Magnoliophyta *Amborella*, several FIBs from monocots, the two detected FIB sequences from *Nelumbo* (early-diverging Eudicot), and FIBs from Caryophyllales, Asterids, and Rosids.

To compare the changes in specific clades and the dynamics of gene expansion following duplication events (small and large scale) undergone by a species, we depict the phylogenomic synteny profiling of FIB proteins per species and the number of proteins per species within a microsynteny community (S9A Fig; S16 Table). In monocots, almost all species contained more than one FIB sequence per genome. One of these remained syntenic but not the others. The exception was those grass species from PACMAD and *Oryza* whose paralogous genes clustered in another community (different genomic context). *Oryza* species are of interest because they all possess two copies of FIB. One copy clustered with the Angiosperm supercluster, while the was other in a specific syntenic block for this genus (we chose six *Oryza* species from different geographic distribution, four of them wild species). No FIB sequence from the sister genus *L*. *perrieri* was found within this *Oryza* community.

In several eudicot species, all their FIB sequences are syntenic in the ancestral Angiosperm supercluster. The Brassicaceae family has experienced two rounds of WGD (At-α and At-β) and a specific WGT in *Brassica* and related genera (Br-α; large-scale duplications), but coupled with small-scale duplication events such as tandems (S10 Fig), especially into the Brassicaceae Lineage I (Camelineae) and Lineage II (Eutremeae and Brassiceae).

Referring to the Tree of Life, paleopolyploidy accounts for plants in the group with more FIB sequences per genome. As an example of FIB duplications by WGD, we inspected *Arabidopsis thaliana* which has three FIB proteins in its genome (AtFIB1-3), located in two different syntenic regions. AtFIB2 is in a syntenic block in chromosome 4, while AtFIB1 and AtFIB3 are both in the same syntenic region of chromosome 5 as tandem duplicated sequences. A synteny approach on FIB proteins among *T*. *hassleriana*, *A*. *arabicum*, and *A*. *thaliana* confirmed the consequences of WGD events on genome structure (S11 Fig).

Among neighboring genes in the Angiosperm supercluster, we detected that five genes remain conserved in FIB in the same genomic context throughout plants: from *Amborella*, passing through monocots, to Rosids. These syntenic conserved genes are a fibrillarin, hydroxyproline-rich glycoprotein, C2H2-like zinc finger protein, MATEefflux family protein, and cytochrome P450, family 715 A1 (S12A Fig; S17 Table). Some genes remained only in *Amborella* and monocots and were lost in eudicots. New genes appear in eudicot clades that are not present in monocots and basal clades. The final group involves a class of genes fixed in all analyzed plants species outside the monocot syntenic block. For genes in the same syntenic block as FIB, we found no functional evidence that correlated to FIB proteins. Details of specific patterns of gene loss and gain on the Angiosperm syntenic block are in S12B–S12E Fig.

### Phylogenomic microsynteny network analysis of animals

**Fig 6:**
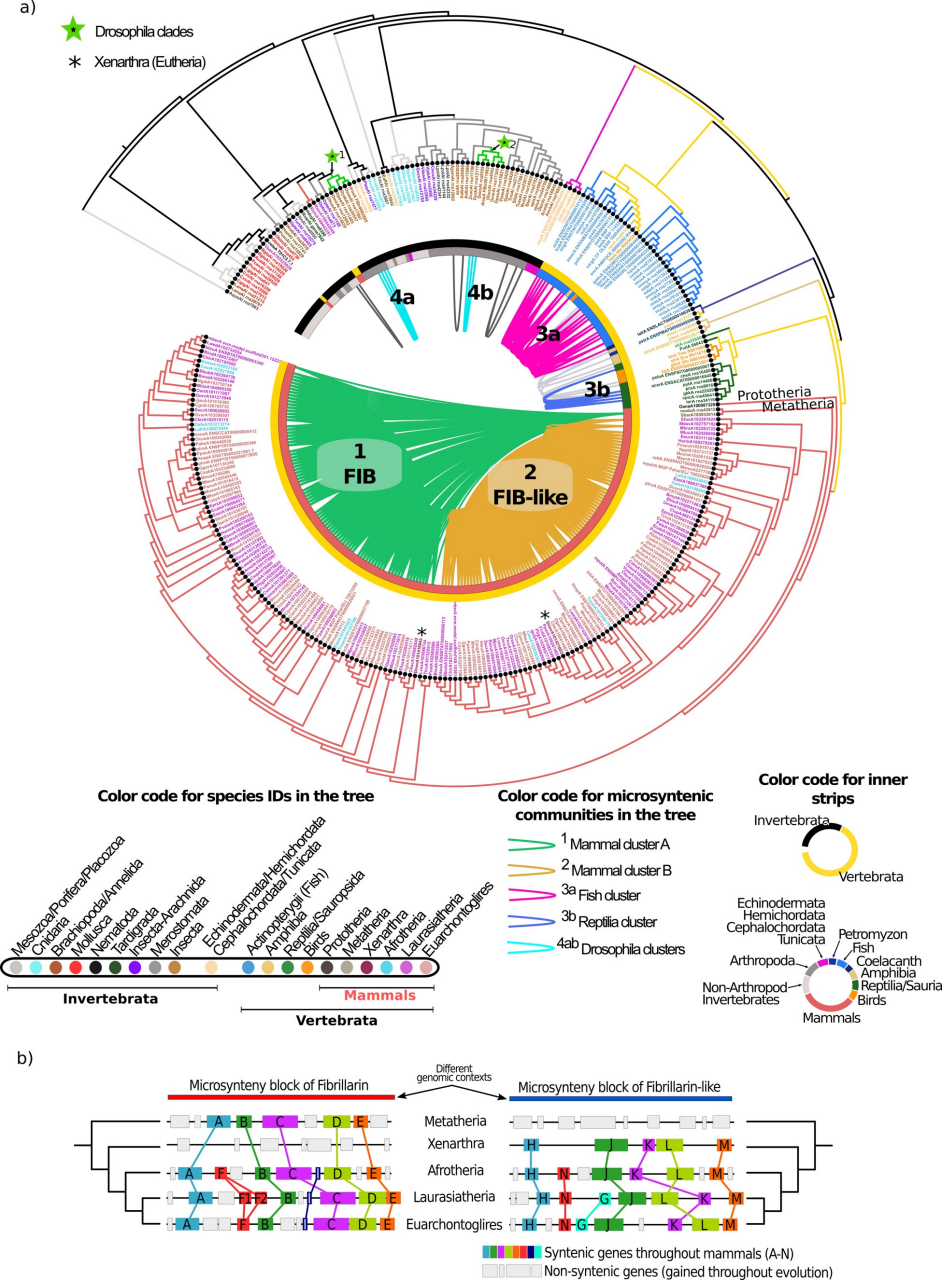
Phylogenomic microsynteny analysis of animal FIB proteins. a) Phylogenetic tree of the total 319 FIB proteins detected across 257 inspected genomes (195 Vertebrata and 62 Invertebrata). Color-coded gene names are on the tree by taxonomic affiliation, as indicated on the left. The color of the first inner strips is by major taxonomic groups: Invertebrata (black) and Vertebrata (yellow). The second inner strips are colored by relevant taxonomic group, as indicated on the left. Internal pairwise connections between tree leaves represent pairwise synteny relationships and are colored by the four detected microsynteny clusters in Vertebrata, as shown in Fig 2B and S13 Fig. Internal pairwise connections in gray represent minor microsynteny relationships not included in any community. Black dots on the tip of the leaves represent genes belonging to the only orthogroup detected in animals. b) Representation of microsynteny blocks of FIB and FIB-like genes. The Xenarthra species *D*. *novemcinctus* is absent in the FIB syntenic block. In the FIB-like syntenic block, only sequences from eutherian mammals are present. Colored blocks represent syntenic genes.

Phylogenetic analysis of 319 animal FIBs showed a clear separation between Invertebrata and Vertebrata and gene clustering per major taxa (Fig 6). Genome sizes from the invertebrate species analyzed ranged from 41 to 2,538 Mb (mean 505.8 Mb). The number of scaffolds in this group ranged from 6 (in the Drosophila model genus) to 331,401 (the gastropoda *Biomphalaria glabrata*), with a mean of 17,569 scaffolds. Animal FIBs belong to a unique orthogroup (black filled circle on the tip of the leaves in Fig 6). The Invertebrata group did not show strong syntenic patterns between FIBs of the 62 genomes from diverse taxa (with very fragmented genomes) analyzed: 14 of 78 FIBs (17%) had a syntenic relationship. Interestingly, Drosophila FIBs (2 FIBs for each of the six species) were clustered in two separate groups on the tree (green stars on the clades of the tree), but each group conserved syntenic relationships between them (Fig 6, clusters 4a and 4b).

In the 195 vertebrate genomes, we detected six microsynteny communities formed by 197 nodes (81.7% of total FIBs were syntenic) and 5868 pairwise syntenic connections (S18 Table). Three of the six syntenic communities were small with particular pairwise connections: the amphibia cluster, the Sauropsida-Reptilia cluster, and a small fish syntenic pair (S13A Fig). After clustering the entire microsynteny network, we detected four distinct communities (S19 Table). The Fish-Reptilia syntenic community (S13B Fig), was split into two specific communities: one for fish and other for reptilia (Fig 6, communities 3a and 3b). The most striking finding was that mammalian FIBs (almost all species have two FIB per genome, as shown in Fig 2E) were divided into two major clusters, as seen in Fig 6. One of these clusters corresponds to classical FIB genes (mammals cluster A in green), and the other to FIB-like genes (mammals cluster B in dark yellow); both of clusters were densely connected with 85 nodes and 3154 edges for FIB cluster, and 79 nodes and 2635 edges for the FIB-like cluster. To gain insights into the genomic context of FIB and FIB-like genes, we retrieved all syntelog pairs within these syntenic blocks. Details of specific patterns of gene loss and gain on the mammal syntenic blocks are in S14 Fig.

**Fig 7:**
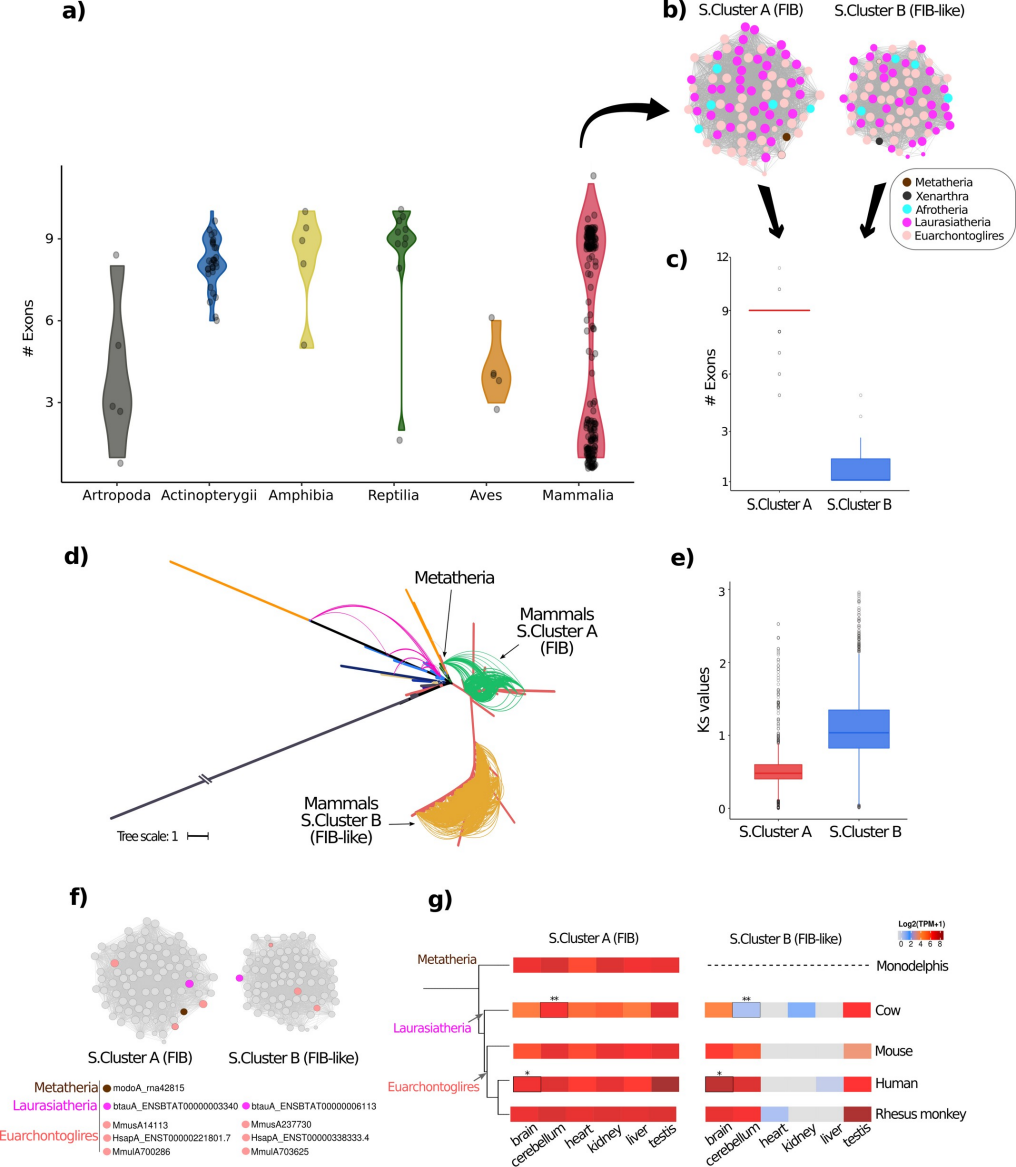
Evolutionary differences between the two microsynteny mammalian clusters. a) Violin plot of the number of exons in each of the five major clades of Chordata animals and arthropods. Points on the plot represent specific data. The number of exons in Actinopterygii, amphibians, and reptiles ranges from 7–10 exons, while that in mammals ranges from 1–10. The curved arrow above the mammalian violin plot indicates the two microsynteny clusters in figure b. b) Two microsynteny clusters detected in mammals and belonging to FIB (cluster A) and FIB-like genes (cluster B) specifically. Arrows under clusters indicate specific boxplots in figure c. c) Boxplot of the number of exons of the genes from the specific microsynteny clusters of mammals. Genes from cluster A (FIB genes) have a mean of 9 exons, while those in microsynteny cluster B (FIB-like genes) have a mean of one exon per gene. d) Depiction of microsynteny communities on a phylogenetic gene tree of animals. The pairwise syntenic relationship of clusters A and B (FIB and FIB-like, respectively) are indicated with black arrows, and the links are colored following Fig 5 to show the absence of syntenic relationship in both clusters (evolving from different genomic context). Green and yellow lines on the tree represent syntenic pairwise connections. e) Ks values for each microsynteny cluster. For the analysis, we carried 1800 and 2664 comparisons of homologous proteins for clusters A and B, respectively. f) Genes chosen from each syntenic cluster were inspected for expression values from transcriptomic atlases (as described in materials and methods). Colored nodes within clusters represent the genes chosen for the analysis. We use genes from species that had two copies, one in each cluster (one FIB and one FIB-like gene), and that had expression information available in the Expression Atlas (EMBL-EBI). g) Heatmap from the expression values of chosen genes in f, clustered according to taxonomy. On the "x" axis (*)frontal lobe, and (**)lung are the tissues used in the analysis.

In FIB sequences from both clusters (mammal A and B; FIB and FIB-like) the number of exons was very variable, ranging from 1 to 11. Thus, we analyzed the number of exons in all Vertebrata clades and a sample of Invertebrata species for outgroup comparison. Almost all species from fish, Amphibia, and Reptilia contain nine exons (Fig 7A). The few fibrillarins from birds ranged from 3 to 6 exons per FIB, while in mammals, the number of exons highly grouped on the shores with no apparent pattern (Fig 7A).

We retrieved and grouped FIBs from each Mammalia synteny community (Fig 7B) and replotted the number of exons per syntenic cluster. Surprisingly, the number of exons is specific to the syntenic community: 82% of FIB genes from the mammals syntenic cluster A contained 9 exons while among the FIB-like genes from mammal cluster B, 56% of genes contained a unique exon and 35% contained 2 exons (summing a 91% of FIBs with one or two exons; Fig 7C). Both microsynteny communities in mammals possess distinctive genomic features that were dependent on their origin. When plotting the syntenic clusters (S.Cluster) on the ML tree (Fig 7D), we found that FIB cluster (S.Cluster A) remains syntenic to *Monodelphis domestica*, a species from the Metatheria clade (S15 Fig). This syntenic link shows evidence that the FIB cluster comes from the Theria clade (e.g., marsupials), and also shows evidence for a duplication event before the Eutheria split (e.g., ruminants, felines, rodents, primates). Therefore, the FIB group remains syntenic to Theria species, unlike the FIB-like group. This finding is in accordance with the number of shared exons between Theria species and members of the mammal cluster A (the group of FIB syntelogs). Furthermore, the FIB-like S.Cluster B (Mammalian B clade) had no syntenic relationship to any Theria species. The branches of both clades of mammals (Mammalian A and B) presented bootstrap values above 70 percent (S15A and S15B Fig).

To better understand the evolutionary divergence of these syntenic communities, we determined the rate of synonymous substitutions (Ks), along with coding sequences (codon alignment strategy) by pairwise comparison of each group of syntelogs (Fig 7E). The distribution of synonymous substitutions among FIB and FIB-like proteins have a different rate of divergence (Ks) according to their genomic context. FIB proteins (S.Cluster A) contained a mean Ks value of 0.52 (Fst_Qu = 0.4 & Trd_Qu = 0.59) compared to FIB-like group (S.Cluster B) with a mean Ks value of 1.1 (Fst_Qu = 0.82 & Trd_Qu = 1.34). The results suggest that FIB syntelogs (S.Cluster A) have a lower rate of substitutions due to the vital role of this form of protein (the ancestral form) and high evolutionary constraints to keep their function. However, the FIB-like syntelogs (S.Cluster B), showed evidence of a high evolutionary rate of nucleotide substitutions, attributed to relaxed evolutionary forces over this duplicated form of protein (derived form) or low evolutionary constraints over this novel form. We used high-quality RNA-seq data available in the Expression Atlas database ([44]; please see Methods) to detect evidence of biased expression of the two different groups of syntelogs (FIB and FIB-like). We selected expression data of FIB and FIB-like proteins from five representative species: one Laurasiatheria, three Euarchontoglires, and one Metatheria species (*M*. *domestica*) within the syntelog groups (Fig 7F). The heatmap of expression data of selected FIBs within a species tree is shown in Fig 7G. We corroborated conserved expression patterns in each group of syntelog FIBs. There is consistent tissue-specific gene expression for FIB genes from the Metatheria species, the Laurasiatheria species, and the three Euarchontoglires species. This data is also in accordance with the low rate of Ks values for S.Cluster A (FIB group) that retains the minor changes in characters to maintain the vital function of FIB along the tree. Moreover, this data could show evidence of gene specialization after duplication of the FIB-like group.

## Discussion

Polyploidy has played a significant role in the evolution of most eukaryotes [28, 45–47]. These duplicated genomic segments (containing genes and regulatory elements) are considered to be a significant force of diversification and provide raw material on which selection can act [27]. Two main principles govern how the genome organized in the eukaryotic nucleus: first by biochemical and functional properties of the chromosomal regions and second by topologically associated domain regions with extensive local chromatin interaction, as recently reviewed [48]. Currently, there are no studies about the consequences of genome duplication on the evolution of this single-copy gene (FIB) through eukaryotic lineages. The study of FIB is relevant due to its vital role in the maintenance of cellular homeostasis and several specific functions. Taking this into account, the microsynteny approach for a well-conserved protein like FIB provides information about the evolutionary maintenance of local sequences as well as conserved functions.

The use of an accurate HMM was critical to detect remote homologues in a set of evolutionarily distant organisms. However, we did not detect homologues of FIB in viruses or any of the 212 bacterial genomes that were analyzed. It is worth noting the presence of FIB, 15.5k (L7Ae), and NOP56 (NOP5) proteins in Archaea but not in Bacteria. The presence of 15.5k and NOP56 homologs in Archaea is of importance for FIB evolutionary history because FIB needs to act in a highly conserved RNP complex [2].

The absence of the GAR domain in Archaeal FIBs correlates with the lack of cellular compartments (as nuclear) because GAR possesses nucleolar signal, which is evolutionarily necessary in eukaryotes [1, 49]. Although Archaea are very similar to Bacteria in many respects [50, 51], some molecular mechanisms in Archaea that are exclusive to eukaryotic organisms (please see [52–55]) as in the case of FIB proteins. This work does not clarify the relationship of the three domains of life, which is out of our scope.

In the last few years, new evidence has shown that FIB is involved in processes such as several types of cancer, viral progression, and bacterial infection response [1]. Earlier experiments carried out in yeast showed that FIB genes from humans and plants could functionally complement the yeast FIB, also referred to as NOP1 [56], demonstrating a high level of FIB conservation throughout eukaryotes. However, earlier results showed neither human nor plant FIB genes could perfectly complement NOP1 in yeast cells, resulting in growth alteration and an aberrant nuclear structure. Therefore, this suggests that the amino acid composition of FIB between lineages is key in specific functions [57].

### Fungi FIBs: A clade-specific synteny pattern

In fungi, compared with animal and plant genomes, paleopolyploidy events occurred to a much lesser extent. Only two ancient whole-genome duplications may have taken place in fungi, one within the Mucoromycotina subphylum before the diversification of the Mucorales order [41], and the other within the Saccharomycetaceae family [45]. Five of the seven sampled species from Mucoromycotina contained FIB duplicates, which might have had their origin from the paleopolyploidization in this lineage.

Comparisons between sequenced fungal genomes often revealed little evidence of extensive interspecific macro- or microsynteny. A short-generation time, asexual reproduction, and lateral gene transfer, among other factors, might have contributed to reductions in synteny conservation within fungi [58]. Indeed, we did not find synteny conservation of FIB homologues among early-diverging lineages, Basidiomycota, and Ascomycota (S7 Fig). However, a mesosyntenic evolutionary pattern, e. g., "conservation of gene content in chromosomes without conservation of gene order and orientation" has been described in Pezizomycotina, especially in Dothideomycetes [58]. This is consistent with our findings of a lack of synteny among FIB homologs within the Dothideomycetes class (except for three Pleosporaceae species) nor in Leotiomycetes (S7A Fig). Further genome sampling would help to resolve this issue.

The species from the Saccharomycetaceae family, which includes *S*. *cerevisiae* yeast, possess many conserved genomic features such as genome size, gene content, and extensive gene collinearity along chromosomes [59, 60]. Accordingly, we found that FIB syntelogs from the F-7 community were very well connected; every node had seven edges each (S6 Fig). This community contained the NOP1 (ScreF) protein from *S*. *cerevisiae*. The ancient WGD and subsequent genome fractionation in some species from this lineage had no repercussions in FIB gene content or genomic context (S7 Fig).

### Plant FIBs: A conserved genomic context on a very shifting genomic history

We observed a single large microsynteny cluster in plants that implies a local genomic region that is less accessible to genomic alterations leading to a higher degree of conservation on these portions of genomic DNA. We detected a conserved genomic context of FIB across plants, even between monocots and eudicots. This conservation was surprising, as plants have undergone several rounds of ancient paleopolyploidization events (ζ, ε, γ, τ, σ, ρ WGD; [47, 61, 62] and several lineage-specific WGD (At-α, At-β, Br-α, Musa-α, Musa-β, among others; [63, 64]. The duplication events resulted in highly fractionated and reshuffled genomes that can lead to several clade-specific syntenic communities. However, this was not the case for FIBs. Recently, [34] carried out a broad microsynteny comparative analysis of all coding genes across 107 plant genomes and found conservation in only 8.7% of all syntenic clusters between monocots and eudicots. The FIB family is within this small percentage of syntenic clusters. Our analysis shows a considerable increase in gene content in taxa that underwent recent specific WGDs as the case of Brassicaceae family, and specifically the *Brassica* genus. Recent work in Glucosinolates (GS) found that gene family expansion has taken place due to the retention of duplicated genes, and most of them (≥70%) are actively expressed in globally or in specific tissues, with different expression patterns [65]. Variation in gene copy numbers, retention of duplicated copies, and posterior sub- neofunctionalization play an essential role in the environmental adaptation and can lead to beneficial or necessary functions as occurs in the salt-tolerant species *E*. *salsugineum* [66]. These results highlight the key role of WGD on gene family expansion and gene functional diversification among plant families. A clear example of results from WGD/WGT is the Brassicaceae genomes; this clade has a mix of duplicated and triplicated regions that occurred after the eudicot paleohexaploidization event (At-γ). These events have played a significant role in Brassicaceae evolution [67, 68]. FIB sequences also accord with these duplication events. In Brassicaceae, two FIB sequences from *A*. *arabicum* (AaFIB's) placed as early branching for the Brassicaceae syntenic sequences, but only one sequence was located on a syntenic block. *A*. *arabicum* belongs to the Aethionemeae tribe, the earliest diverging clade from the rest of the Brassicaceae family (core Brassicaceae), which harbors many genes not found in duplicated syntenic blocks as with the rest of the core Brassicaceae [69–71]. As expected, all the Brassicaceae species contained two duplicated syntenic blocks, except for *B*. *rapa*, which contained four genes in different duplicated syntenic blocks due to their specific Br-a duplication event. Even *A*. *arabicum* presented a second syntenic block on Scaffold 136, but this lacked duplicated FIB sequence most probably due to the normal process of fractionation (homeologous gene loss). Previously, [33] some communities were found to transpose to another genomic context that led to new functions with amino acids substitution rates due to a different location in the genome.

### Avian FIBs: A still intriguing case of genomic absence

Birds present novel functional characteristics only present in its apomorphic clade such as wings, feathers, lightweight bones, and an exclusive excretory and urinary system [72, 73], making this group a very interesting clade from an evolutionary point of view [74]. Birds tend to lack several essential proteins for life, and FIB is one of these missing proteins [75]. Prior research [75] found that birds lack approximately 274 proteins present in syntenic regions in most of the Vertebrata lineages. Many of these missing proteins are associated with vital functions in mammals, physiology of organs and systems in mammals, lethality, and genetic disorders.

Several novel paralogs in avian species were identified that could provide compensation for vital physiological functions and relevant pathways for this clade. A previous [76] RNA-seq analysis on birds showed that several missing genes were present in most avian species. They found that most of the genes correspond to CG-biased genome regions, the most difficult to sequence, assemble and annotate. They retrieved 91 of the 274 genes previously reported as missing [75]; GC content is the primary cause of miss-assembled bird genomes, and novel technologies of sequencing that do not rely on PCR can improve the assembly and annotation of avian genomes [77]. Although [76] found several missing genes, there are still several genes missing like FIB in avian genomes. In this study, we found that birds lack FIB genes in almost all of the 53 analyzed genomes. There were five exceptions to this rule, and these presented a distinctive protein composition when compared to FIBs from other eukaryotic organisms; FIBs from birds lack the GAR domain and space region, regressing to an Archaea likeness. Considering that the GAR domain is essential for nuclear localization and lack of this domain may indicate a new localization with a different role for this protein in the few bird species that have it. The genomic analysis is relevant as previous experiments using antibodies for immunolocalization in *G*. *gallus* cells may have produced misleading results [78], as there is no genomic FIB in this species. Western blot analysis from Arabidopsis, human, and chicken was carried out and showed a corresponding band for FIB in plants and humans but not in chicken (S16 Fig). However, there still the possibility that chicken FIB does not cross-react with the antibody used.

From invertebrate to vertebrate animals (with the exclusion of mammals), all species retained only one FIB sequence per genome. Some exceptions, like the Mollusca clade, some insects, and especially the genus Drosophila. However, the case of mammals is of particular interest. In this work, we detected clear evidence that mammals have undergone a duplication of these proteins after the Theria split so that the new Eutheria clade has two copies of FIB. One of this copies retained the ancient functions (FIB), as shown by the syntenic analysis, but the newly formed copy (FIB-like) has evolved independently in its new genomic context and has been shaped to perform specific unknown functions, as evidenced by the expression analysis of both copies (Fig 7).

### Mammals FIBs: An ancient duplication event on a very conserved genomic context

Polyploidy is rare in animals, even though there are several examples of insects and vertebrate animals (mainly fish and amphibians) that have undergone WGD [79]. Polyploidy has played a central role in the expansion of individual protein families and [80] has provided evolutionary opportunities for the success of the species.

What determines the rate of protein sequence change is a central question for understanding molecular evolution. Several studies have reported different determinants that can influence dN/dS, such as functional relevance of a protein, its expression among tissues, pleiotropy, protein-protein interaction, and secondary structure [81, 82].

From the 1,552,319 species of animals, invertebrates represent about 95% of all species [83, 84]; 1,242,040 species belong to Arthropoda (~80% of total animals), and of these, 1,020,007 species belong to the Insecta clade (~66% of the total) [83]. Most of the roughly sequenced genomes for Invertebrata correspond to Arthropoda or Nematoda because of their importance for human health, because they are pest species, or because they are model species for elementary development biology [84]. The significant level of diversity in this group and the lack of more genome sampling per taxonomic group is a current limitation to find a deep syntenic relationship in this major lineage. Nevertheless, invertebrate FIBs kept as a well-defined clade next to vertebrate FIBs. Further research would be required to address this group in particular. Our results show a clear division of two different FIBs in Mammals. These findings are surprising since there is a wealth of research on human FIB, and several different pathways are known. However, some known activities may involve the second FIB (FIB-like protein; [1]). Currently, no commercial antibodies exist that can distinguish between the two paralogues proteins. Therefore, it is pertinent to define the specific role of each of these proteins in mammalian cells. Our analysis points to further functional studies on the second mammalian FIB from which there is no published information, and all studies that rely on antibodies are unable to differentiate between the two genes. Considering that FIB or FIB-like genes can be involved in different processes like the formation of specialized ribosomes for particular translation initiation involved in tumor progression [15, 85], sensors for bacterial infection [14] and some viral progression processes [86]. Further studies are required to define their specific role.

As mentioned above, mammals have undergone fewer events of WGD in comparison to plants. Two ancient rounds of WGD, termed as 2R, in the basal branch of mammals, are driving the genome rearrangements in this clade [87, 88]. In a study on 87 complete sequenced genomes, [34] found that a large proportion of single-copy genes in mammals are in significant microsynteny clusters (genes that remain syntenic across almost all analyzed species), and the lineage-specific microsynteny communities (specific transposition in mammals) were genomic outliers. These outliers, or rebel genes (as termed in [34]), are of particular interest because they can potentially contribute to trait and lineage evolution. Therefore, transposed genes to a new genomic context can lead to new mechanisms of molecular evolution, as seen in the FIB-like group.

Detecting long-term conservation and lineage-specific dynamics of genomic characters by microsynteny approach can help to understand the phenotypic traits and functional dynamics of genes. This study shed new light into FIB dynamics trough out the Tree of Life, especially into significant groups of Eukarya. The results can direct functional and fundamental questions about the structure, composition, and behavior of FIBs according to the evolutionary history of this small but essential family of proteins.

## Materials and methods

### Genome databases searches and sequences retrieval

For the analysis of the three domains of life, we selected the genomes of 212 Bacteria, 148 Archaea, 75 protist, 157 fungi, 153 plant *sensu lato* (140 plants and 13 algae), and 257 animals (62 invertebrata and 195 vertebrata). We also search for 47 giant viruses and viruses that infect Bacteria and Archaea (S1 Table). We annotated the three giant uncultured marine viruses from environmental samples by using Prokka v1.14.5 [89] and we set parameters as follow:—kingdom Viruses—addgenes—mincontiglen 200—evalue 0.001—locustag UncMarV[123]. In the case of plants and animals, we retrieved all available genomes (completely assembled at chromosome or scaffold level); due their large genome size, there are not as many available as for organisms with small-size genomes (e.g., Bacteria, Archaea, fungi, some protist). For Bacteria, Archaea, fungi, and protist (which have several assemblies each due their reduced genome size), we selected well sequenced representative species from all major and minor clades to cover all the biological diversity (from different subphylum, order, class, family, and genus). Different databases where used such as Phytozome [90], ENSEMBL [91], NCBI (https://www.ncbi.nlm.nih.gov/), GigaDB (http://gigadb.org/), MycoCosm ([92]). For bacteria, we considered 30 species from the candidate phyla radiation (CPR) group, spanning the nine major subgroups reported in [37]. As an exploratory analysis, from the 777 assemblies of the CPR group reported in [36], we only analyzed the genome of one representative strain per species because many different assemblies are reported per species (e.g., 197 different assemblies for Parcubacteria group bacterium GW2011; S2 Table). We built a Hidden Markov Model (HMM) from the fibrillarin domain (Pfam: PF01269) of 450 unique fibrillarin sequences retrieved from a psi-blast analysis (3-iterations in each search; [93]) against the nr database from the NCBI and the refseq-protein databases from Fungi (taxid:4751), Alveolata (taxid:33630), Rhizaria (taxid:543769), Amoebozoa (taxid:554915), Bacteria (taxid:2), and Archaea (taxid:2157). For these searches, considering that fibrillarin is highly conserved across lineages (from Archaea to Eukarya [1, 2]), the queries were the human FIB protein (NP_001427.2) and the AtFIB2 protein from *A*. *thaliana* (NP_567724.1), as these proteins are functionally well characterized [94–96]. We use this model to detect fibrillarin sequences on the 1049 selected genomes across the three domains of life. On the other hand, and following the same strategy as for fibrillarin, we built a HMM for the 15.5k protein (L7Ae homolog in Archaea) and for the NOP56 protein (NOP5 in Archaea), because these two protein are part of the snoRNP complex that interacts directly with FIB [2, 3]. The HMMER package v3.1b2 [97] used to build the all the models and to perform the searches on the selected genomes.

After the searches with our HMM-FIB model, we detected fibrillarin sequences by using the EMBOSS suit [98]. All the fibrillarin sequences were manually checked to discard truncated sequences and non-fibrillarin sequences. All retrieved FIB sequences were annotated by SUPERFAMILY database v1.75 [99], HMMER database (https://www.ebi.ac.uk/Tools/hmmer/), and Pfam database (http://pfam.xfam.org/). The GFF and BED annotation files of each genome used to discard isoforms, and the longest gene version taken for the analyses. The FIB sequences aligned by hmmalign tool of HMMER package [97] using our HMM-FIB model, and then the GAR domain was separately aligned in UGENE v.1.9.8 [100] by using Muscle [101]. The complete set of analyzed genomes in Fig 1A was depicted by using suburstR package [102] in R v3.4.1 [103].

### Phylogenetic analyses and species tree

Before the phylogenetic analysis, evaluation of the global amino acid alignment was done in Prottest v.3.4.2 [104] to find the best empirical substitution model. We built four separately ML phylogenetic trees for each set of organisms (one for plants [JTT substitution model], one for animals [VT model], one for fungi [WAG model], and another one for all the fibrillarins sequences from the three domains of life [LG model]). The software RAxML v8.2 [105] used to build the phylogenetic trees using the bootstopping option “-# autoMRE” and an empirical base frequencies. For the species trees, we used the species taxid from Taxonomy Common Tree tool of NCBI (https://www.ncbi.nlm.nih.gov/) as input for the tree reconstruction with the ETE toolkit [106]. The trees were visualized and annotated in iTOL v4 [107].

### Microsynteny network approach

For this approach, we followed a previously reported pipeline [108, 109], which consists of synteny block calculations among diverse genomes, network constructions, and detection of dense syntenic communities for one or more gene families (https://github.com/zhaotao1987/SynNet-Pipeline). Briefly, we conducted reciprocal all-against-all pairwise protein comparisons (inter- and intra-genomic comparisons) of selected lineages by using RAPSearch2 software [110] and setting parameters as follows: “-z 10 -b 0 -v 20 -t a -a t”. These searches were performed separately for fungal (157), plant (153), invertebrata (62), and vertebrata genomes (195). We performed *n2* times comparisons of annotated genomes (*n* stands for the number of species analyzed), and then performed *n*(*n*+1)/2 synteny block detection using MCScanX software [111]. The comparison files and gene position files (GFF/BED) generated were used to detect pairwise synteny blocks utilizing MCScanX tool using default parameters and creating a score matrix of all syntenic relationships inside the studied lineages. We modified these score matrix files to a two-column tabular format obtaining three big “Final network files” (one for fungi, one for plants, and another one for animals) where all pairwise inter- and the intra-species syntenic relationship of the complete analyzed genomes was contained. Then we used the IDs of the genes detected by our HMM-FIB model to retrieve all the syntenic information of FIB family from these “Final network files” for each analyzed group. The synteny information of FIB family used to construct densely connected clusters by using the Clique percolation method (k-clique = 3) implemented in CFinder [112, 113]. The resulting syntenic communities were visualized in Cytoscape v3.5.1 [114] and Gephi v0.9.1 [115]. Finally, visualization of all the information of the syntenic communities in their respective phylogenetic gene trees (phylogenetic profiling method). For these Trees, we included all genes found with our HMM-FIB model, including those genes without synteny relationship (no syntenic information).

Genome comparisons and microsynteny analyses in the S10 and S11 Figs were carried out using the comparative genomic tools SynFind and GEvo from CoGe [116]. To run SynFind we set the parameters as follows: Comparison algorithm: Last [117], gene windows size: 40, a minimum number of genes: 4, scoring function: collinear. For GEvo microsynteny analysis, the parameters were set as default.

### Molecular evolution

The protein sequences and their corresponding coding sequences were aligned and converted into codon alignments using ParaAT v2.0 [118] coupled to KaKs_Calculator 2.0 program [119] for the analysis of nonsynonymous (Ka) and synonymous (Ks) rates (Ka/Ks) of each codon alignment. Ks values were computed for all possible pairwise combinations of the 60 (1770 combinations) and 73 (2628combinations) codon aligned syntelogs from mammal cluster A (FIB) and mammal cluster B (FIB-like), respectively.

### Gene expression analysis

For the Fibrillarin gene expression analysis, we retrieved information from the Expression Atlas database ([44]; https://www.ebi.ac.uk/gxa/home). The expression sets ID were: E-MTAB-3716 (Human), E-MTAB-3719 (Monodelphis), E-MTAB-3718 (mouse), E-MTAB-3717 (Rhesus monkey), and E-MTAB-2798 (cow). Heatmaps in Fig 7G was generated with ComplexHeatmap package [120] from Bioconductor project [121].

## Supporting information

S1 Fig Phylogenetic tree of the total 1067 FIB proteins found in Archaea and Eukarya, colored according to main taxonomic groups. The tree root was placed between the clade leading to Archeae and the Eukaryota. The domain regions of the total 1067 fibrillarin proteins were aligned with MUSCLE v3.8.31 [101]. The alignment was trimmed with TrimAl v3.8.31 with the [-automated1] option, remaining a total of 202 positions in the final alignment. Phylogenetic inference was performed with RaxML v8.2.12 [105] with the LG+F model and a total of 500 bootstraps repetitions, determined by the bootstopping criterion, i.e. the [-autoMRE] option. Finally, the tree was visualized in ITOL [107]. Colored branches of fish sequences only include Actinopterygii.
(PDF)

S2 Fig FIB model and sequence alignment of FIB proteins from different lineages spanning Archaea and Eukarya. a) Depiction of the structure of FIB protein made from the alignment of diverse lineages of Archaea and Eukarya. b) Sequence alignment of FIB proteins from different lineages of Archaea and Eukarya.
(PDF)

S3 Fig Phylogenetic tree of FIB proteins from protists. The number of exons is to the right of each leaf label with orange circles, whose sizes are proportional to the number of exons. The explicit number of exons is inside each circle. The total 103 protist sequences were aligned with hmmalign to a custom HMM-FIB model with hmmalign in HMMER3 3.1b2 [97]. The C-terminal region outside the FIB domain was removed and the N-terminal region (containing the GAR sequence) was independently aligned with MUSCLE v3.8.31 [101] in UGENE v1.31.0 [100]. The resultant alignment was trimmed with TrimAl v1.2rev59 with the [-automated1] option v3.8.31, thus, the final alignment consisted of 174 sites. Phylogenetic inference was performed with RaxML v8.2.12 [105] using the LG+F model (best fitted for these data) and 600 bootstrap replicates, determined by the bootstopping criterion, i.e. [-autoMRE] option. Finally, the tree was visualized in ITOL [107]. Labels of tree leaves colored according to main taxonomic groups as indicated in the legend.
(PDF)

S4 Fig Phylogeny of fungal species and their FIB proteins. Phylogenetic relationships of the 157 fungal species for FIB proteins in the present study. Tree branches are not at scale and only depict the species relationships (topology). The species tree was initially constructed based on the NCBI taxonomy IDs (each species TaxID is indicated after a dash ‘-’ in its corresponding label name) with ETE 3 v3.1.1 [106] and visualized in ITOL 4.2.3 [107]. The tree was manually modified to fit the cladogram of the Fungi kingdom proposed by [39]. Relevant taxonomic groups in internal nodes and branches. A cross in a branch leading to a species name indicates a possible loss of the FIB protein in that species, and a circle indicates two or more FIB duplicates. To the right of the tree a presence/absence matrix (color filled figures [presence], open figure [absence]) indicating the presence of synteny communities in each species is depicted (numbered and colored as in S6 Fig. Communities belonging to Ascomycota, Basidiomycota, and Mucorinae are depicted as squares, circles, and stars, respectively. Information regarding the species abbreviations used in the present study and the number of FIB proteins is presented.
(PDF)

S5 Fig Phylogeny of FIB proteins from fungi and exon number per sequence. The tree was rooted in the branch leading to the Microsporidia clade. The number of exons to the right of each leaf label with orange circles, whose size is proportional to the number of exons. The explicit number of exons inside each circle. The total 170 fungal FIB protein sequences were firstly aligned to a custom HMM-FIB model with hmmalign in HMMER3 3.1b2 [97], the C-terminal region outside the FIB domain was removed, and the N-terminal region (containing the GAR sequence) was independently aligned with MUSCLE v3.8.31 [101] in UGENE v1.31.0 [100]. The resultant alignment was trimmed with TrimAl v1.2rev59 with the [-automated1] option v3.8.31. The final alignment consisted of 288 sites including the FIB and GAR domains. Phylogenetic inference was performed with RaxML v8.2.12 [105] using the WAG+I+F model (best fitted for these data) and 500 bootstrap replicates, determined by the bootstopping criterion, e.g. [-autoMRE] option.
(PDF)

S6 Fig Synteny relationships of the fungal FIB homologues. Nine synteny network communities found at k-clique = 3. Nodes represent fungal FIB proteins and edges represent pairwise synteny relationships. Nodes sizes are proportional to the number of synteny connections they share. Nodes marked with black arrows indicate XhveF_KZF19727 and BpnaF_EMC92328 FIB proteins of Xylona heveae and Baudoinia panamericana, respectively. *Only comprises Metschnikowiaceae and Debaryomycetaceae families within Saccharomycetales; **Only comprises Auriculariales and Polyporales orders within Agaricomycetes (see S2 Fig).
(PDF)

S7 Fig Phylogeny of fungal species and their FIB proteins. a) Phylogenetic relationships of the 157 fungal species for FIB proteins in the present study. Tree branches are not at scale and only depict the species relationships (topology). The species tree was initially constructed based on the NCBI taxonomy IDs (each species TaxID is indicated after a dash ‘-’ in its corresponding label name) with ETE 3 v3.1.1 [106] and visualized in ITOL 4.2.3 [107]. The tree was manually modified to fit the cladogram of the Fungi kingdom proposed by [39]. Relevant taxonomic groups in internal nodes and branches. A cross in a branch leading to a species name indicates a possible loss of the FIB protein in that species, and a circle indicates two or more FIB duplicates. To the right of the tree a presence (closed figure)-absence(open figure) matrix indicating the presence of synteny communities in each species is depicted (numbered and colored as in S6 Fig). Communities belonging to Ascomycota, Basidiomycota, and Mucorinae depicted as squares, circles, and stars, respectively. Information regarding the species abbreviations used in the present study and the number of FIB proteins also presented. b) Microsynteny clusters in fungi. Eight communities were clustered and colored according S6 Fig.
(PDF)

S8 Fig Networks of synteny blocks containing fungal FIB homologues. Network representation of the protein-coding genes contained within the same synteny block indexes as fungal FIB homologues. Nodes represent proteins and edges represent pairwise synteny relationships. Node sizes are proportional to the number of synteny connections per node (degree), however these sizes are not comparable among independent networks. To construct these networks, we retrieved all pairwise relationships (edges) between proteins (nodes) that matched the same block indexes (indicated in S11 Table) as the fungal FIB homologues found within each of the nine fungal FIB communities (S6 Fig). Then, we used CFinder at k-clique = 3 to find communities of synteny homologous proteins; the original fungal FIB communities were also recovered (S6 Fig). For easier visualization, communities with low number of nodes were filtered out and we only depict communities with a determined number of nodes or above (indicated by ‘AF’ in the figure; the applied filter was arbitrarily chosen for each network). Colors were set to help define each community. The complete sets of nodes and edges, before and after CFinder analysis are listed in S11 and S12 Tables, respectively. Taking into the account the number of syntelogs but not the number of species, the biggest syntenic block corresponded to the F-4 FIB community, which was composed of at least 40 syntelogs from the Ustilaginaceae family (S8D Fig). The smallest syntenic blocks, taking into account both the number of syntelogs and the number species, were F-3, F-8, and F-9 (S8C, S8H and S8I Fig). F-1, F-6, and F-7 were the biggest syntenic blocks when number of syntelogs and number of species were taken into account (S8A, S8F and S8G Fig). Abbreviations used in the networks: Original FIB community (FC); total number of communities at k-clique = 3 (TC); range of number of nodes per community at k-clique = 3 (RN); number of communities depicted (CD); and applied filter (AF, the minimum number of nodes per community). a) F-1 (Eurotiomycetidae), b) F-2 (Chaetothyriales), c) F-3 (Mucorineae), d) F-4 (Ustilaginacae), e) F-5 (Saccharomycetales), f) F-6 (Sordariomycetes), g) F-7 (Saccharomycetaceae), h) F-8 (Agaricomycetes), and I) F-9 (Pleosporaceae).
(PDF)

S9 Fig Phylogeny of plant species and their FIB proteins. a) Phylogenetic relationships of the 328 plant species for FIB proteins in the present study. Tree branches are not at scale and only depict the species relationships (topology). The species tree was initially constructed based on the NCBI taxonomy IDs (each species TaxID is indicated after a dash ‘-’ in its corresponding label name) with ETE 3 v3.1.1 [106] and visualized in ITOL 4.2.3 [107]. To the right of the tree a presence (closed figure)/absence(open figure) matrix indicating the presence of synteny communities in each species is depicted (numbered and colored as in Fig 5). b) Microsynteny clusters of the total 223 FIB proteins from plants. Six communities were clustered according the clique = 3 to find dense communities of synteny homologous proteins.
(PDF)

S10 Fig Colored lines into the tree represent pairwise tandem relationship. Colors of lines used only for easy visualization and has not special meaning. Grey lines connections into the tree represent the syntenic communities showed in Fig 5. The color-coded names of genes on the tree are according to their taxonomic affiliation as indicated on the right. Inner strips colored according to major taxonomic groups: algae (red) and angiosperms (green). Black filled circles on the tip of the leaves represent genes belonging to the unique orthogroup detected in plants. Yellow starts inside the black filled circles represent genes expanded by tandem duplication and yellow stars on the nodes of the tree (only two) represent clades that expanded by tandem duplication events. The total 328 plant FIB protein sequences were firstly aligned to a custom HMM-FIB model with hmmalign in HMMER3 3.1b2 [97], the C-terminal region outside the FIB domain was removed, and the N-terminal region (containing the GAR sequence) was independently aligned with MUSCLE v3.8.31 [101] in UGENE v1.31.0 [100]. Phylogenetic inference was performed with RaxML v8.2.12 [105] using the JTT+I+F model (best fitted for these data) and 500 bootstrap replicates, determined by the bootstopping criterion, e.g. [-autoMRE] option.
(PDF)

S11 Fig Microsynteny analysis between *A*. *thaliana* (At), *A*. *arabicum* (Aa), and *T*. *hassleriana* (Th) species. The synteny analysis show the consequence of the WGD and the different fractionation patterns in each group. *A*. *arabicum*, the early-branching of the rest of Brassicaceae, contains only one FIB protein in comparison to *A*. *thaliana* that host three FIB proteins into two duplicated blocks (one of them [AtFIB3] in Chr5 created by tandem duplication). *T*. *hassleriana*, from the Cleomaceae sister family for Brassicaceae, has undergone an independent genome triplication (Th-α), which raise three syntenic blocks in comparison to *A*. *arabicum*. Colored lines indicate syntenic relationship of FIB genes between syntenic blocks. Chr = chromosome. The analysis can be regenerated in http://genomeevolution.org/r/numm.
(PDF)

S12 Fig Schematic representation of syntenic genes surrounding FIBs on the Angiosperm syntenic blocks. a Depiction of the microsynteny networks of the five genes that were conserved through plant linage. b) Depiction of syntenic genes shared only by amborella and monocots. These genes were not found in eudicots. c) Depiction of syntenic genes that were conserved through amborella, monocots and basal eudicots, but not the core eudicots. d) Depiction of syntenic genes that were shared only by Rosids, but not other eudicots nor monocots. These genes were probably gained in eudicot evolution. e) Depiction of syntenic genes shared by almost all angiosperms but lost in the syntenic blocks of monocots. f) Depiction of the total of genes found into the syntenic blocks of FIB genes. Communities above k-clique = 2 were not depicted due its large amount of genes in this category.
(PDF)

S13 Fig Phylogeny of animal vertebrata species and their FIB proteins. a) Phylogenetic relationships of the 195 animal species for FIB proteins in the present study. Tree branches are not at scale and only depict the species relationships (topology). The species tree was initially constructed based on the NCBI taxonomy IDs (each species TaxID is indicated after a dash ‘-’ in its corresponding label name) with ETE 3 v3.1.1 [106] and visualized in ITOL 4.2.3 [107]. To the right of the tree a presence (closed figure)/absence(open figure) matrix indicating the presence of synteny communities in each species is depicted (numbered and colored as in Fig 5). b) Microsynteny clusters of the total 197 syntenic FIB proteins from vertebrates. Six communities were clustered and coloring according Fig 6.
(PDF)

S14 Fig Schematic representation of syntenic genes surrounding FIB and FIB-like genes on mammalian syntenic blocks. a) Depiction of microsynteny networks of genes into the “FIB microsynteny block”, that were conserved through mammalian species, but not the eutherian *D*. *novemcinctus*. Only microsynteny networks containing the metatherian *M*. *domestica* are shown. b) Depiction of microsynteny networks genes into the “FIB-like microsynteny block”, that were conserved through eutherian mammals. Only microsynteny networks containing the Xenarthra *D*. *novemcinctus* are shown. FIB and FIB-like synteny blocks remains in different genomic contexts throughout mammalian evolution.
(PDF)

S15 Fig Phylogenomic microsynteny analysis of animal FIB proteins. a) Phylogenetic tree with branch lengths of the total 319 detected FIB proteins shown the major clades of mammals (FIB and FIB-like). b) Phylogenetic tree with branch lengths of the total 319 detected FIB proteins and showing the syntenic connections of mammal clusters. The FIB cluster remains connected to basal Theria groups, showing evidence of ancient block conservation of this group. FIB-like cluster (yellow links) transposed to another genomic context but remains syntenic in eutheria mammals. Color-code of the names of genes on the tree are according Fig 6.
(PDF)

S16 Fig Western blot analysis of fibrillarin on selected organism. We decided to test if birds lack FIBs as the genomic data suggest. We carried out extracts from the whole *Arabidopsis thaliana* plant, heart tissue from *Gallus gallus domesticus*, as a representatives of the avian clade, and extract from human cells (HeLa cells), as a representative of Mammals. We used the commercial antibody from abcam ab166630.
(PDF)

S1 Table List of the 47 viral genomes that were analyzed in the search for FIB-like proteins. (XLSX)

S2 Table List of the 212 bacterial genomes that were analyzed in the search for FIB proteins. (XLSX)

S3 Table Sequences retrieved in the CPR group of Bacteria by using the HMM-FIB model. No FIB sequences were detected into this group, but members of the methyltransferase superfamily.
(XLSX)

S4 Table Results of the search of 15.5k and NOP56 proteins (part of the box C/D snoRNA, together with FIB) and RG-rich regions into Bacterial and Archaeal proteomes. HMM models for 15.5k and NOP56 were used for the searches into the 212 proteomes of Bacteria and the 148 proteomes of Archaea. We also used three different HMM models from the characteristic RG-rich region of FIB (the gar domain) and the RG-rich regions from gar protein (the gar1 and gar2 boxes). The three models for the GAR regions were taken from Guillen-Chable *et al*. Cells. 2020; 9(1143).
(XLSX)

S5 Table List of the 148 archaeal genomes that were analyzed in the search for FIB proteins. (XLSX)

S6 Table List of the 76 protist genomes that were analyzed in the search for FIB proteins. (XLSX)

S7 Table List of the 157 fungal genomes that were analyzed in the search for FIB proteins. (XLSX)

S8 Table List of the 153 plant genomes that were analyzed in the search for FIB proteins. (XLSX)

S9 Table List of the 257 animal genomes that were analyzed in the search for Fibrillarin proteins. (XLSX)

S10 Table Total of the FIB protein sequences analyzed in this work. (XLSX)

S11 Table Microsynteny Network of the FIB proteins found in the fungal genomes. This network was formed by all pairwise syntenic genes (Node1-Node2) found by MCScanX software.
(XLSX)

S12 Table Fungi Microsynteny communities found at k-clique = 3 by Cfinder. (XLSX)

S13 Table All edges found at k-clique = 3 for the protein-coding genes within the Fungi FIB syntenic blocks. (XLSX)

S14 Table Total number of proteins (nodes) found in the same syntenic blocks as fungal FIB homologues. Annotations were made with Blas2GO against the refseq collection of fungal protein sequences.
(XLSX)

S15 Table Microsynteny Network of the FIB proteins found in the plant genomes. This network was formed by all pairwise syntenic genes (Node1-Node2) found by MCScanX software.
(XLSX)

S16 Table Plant Microsynteny communities found at k-clique = 3 by Cfinder. (XLSX)

S17 Table Total number of proteins (nodes) found in the same syntenic blocks as plant FIB homologues. These networks were formed by all pairwise syntenic genes (Node1-Node2) retrieved from the “collinearity” files produced by MCScanX software. The annotations of the proteins were retrieved from the genome metadata (annotations files) from each specie.
(XLSX)

S18 Table Microsynteny Network of the FIB proteins found in the Animal genomes. This network was formed by all pairwise syntenic genes (Node1-Node2) found by MCScanX software.
(XLSX)

S19 Table Animal Microsynteny communities found at k-clique = 3 by Cfinder. (XLSX)
