# Supplementary figures and images for: Fibrillarin evolution through the Tree of Life: Comparative genomics and microsynteny network analyses provide new insights into the evolutionary history of Fibrillarin
Source: PLoS Comput Biol. 2020 Oct 19;16(10):e1008318. doi: 10.1371/journal.pcbi.1008318 (PMC7608942; doi:10.1371/journal.pcbi.1008318)

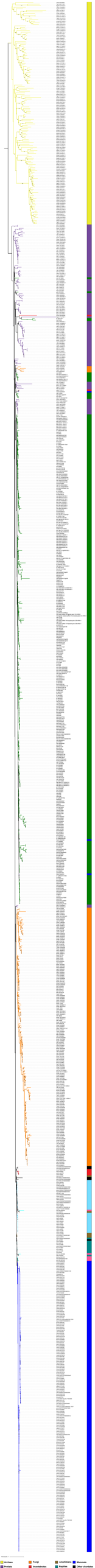

S1 Fig

Supplement: S1 Fig — The tree root was placed between the clade leading to Archeae and the Eukaryota. The domain regions of the total 1067 fibrillarin proteins were aligned with MUSCLE v3.8.31 [101]. The alignment was trimmed with TrimAl v3.8.31 with the [-automated1] option, remaining a total of 202 positions in the final alignment. Phylogenetic inference was performed with RaxML v8.2.12 [105] with the LG+F model and a total of 500 bootstraps repetitions, determined by the bootstopping criterion, i.e. the [-autoMRE] option. Finally, the tree was visualized in ITOL [107]. Colored branches of fish sequences only include Actinopterygii. (PDF) [file pcbi.1008318.s001.pdf]

**b)**

### Methyltransferase domain (MTase)

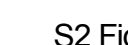

Supplement: S2 Fig — a) Depiction of the structure of FIB protein made from the alignment of diverse lineages of Archaea and Eukarya. b) Sequence alignment of FIB proteins from different lineages of Archaea and Eukarya. (PDF) [file pcbi.1008318.s002.pdf]

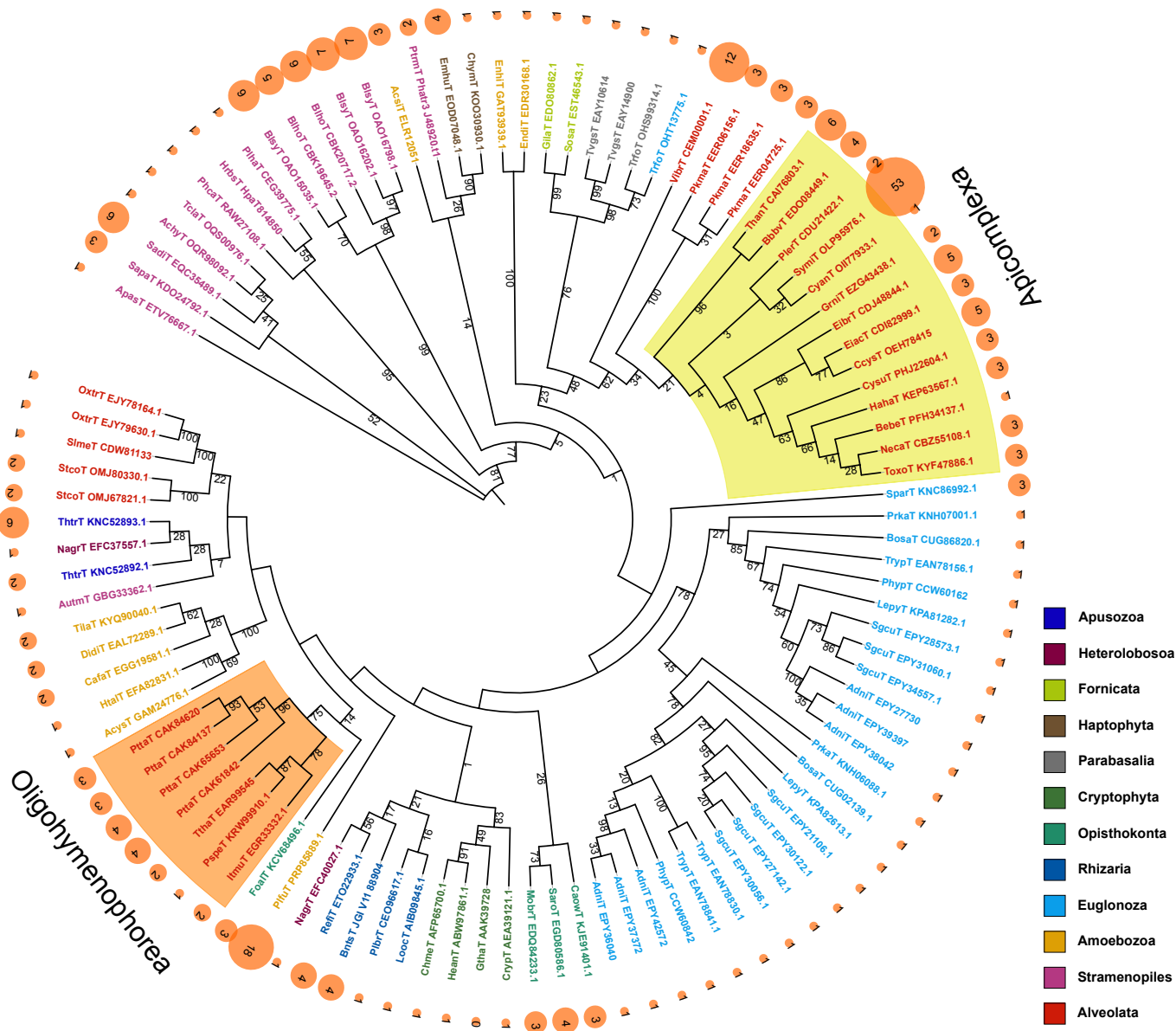

S3 Fig

Supplement: S3 Fig — The number of exons is to the right of each leaf label with orange circles, whose sizes are proportional to the number of exons. The explicit number of exons is inside each circle. The total 103 protist sequences were aligned with hmmalign to a custom HMM-FIB model with hmmalign in HMMER3 3.1b2 [97]. The C-terminal region outside the FIB domain was removed and the N-terminal region (containing the GAR sequence) was independently aligned with MUSCLE v3.8.31 [101] in UGENE v1.31.0 [100]. The resultant alignment was trimmed with TrimAl v1.2rev59 with the [-automated1] option v3.8.31, thus, the final alignment consisted of 174 sites. Phylogenetic inference was performed with RaxML v8.2.12 [105] using the LG+F model (best fitted for these data) and 600 bootstrap replicates, determined by the bootstopping criterion, i.e. [-autoMRE] option. Finally, the tree was visualized in ITOL [107]. Labels of tree leaves colored according to main taxonomic groups as indicated in the legend. (PDF) [file pcbi.1008318.s003.pdf]

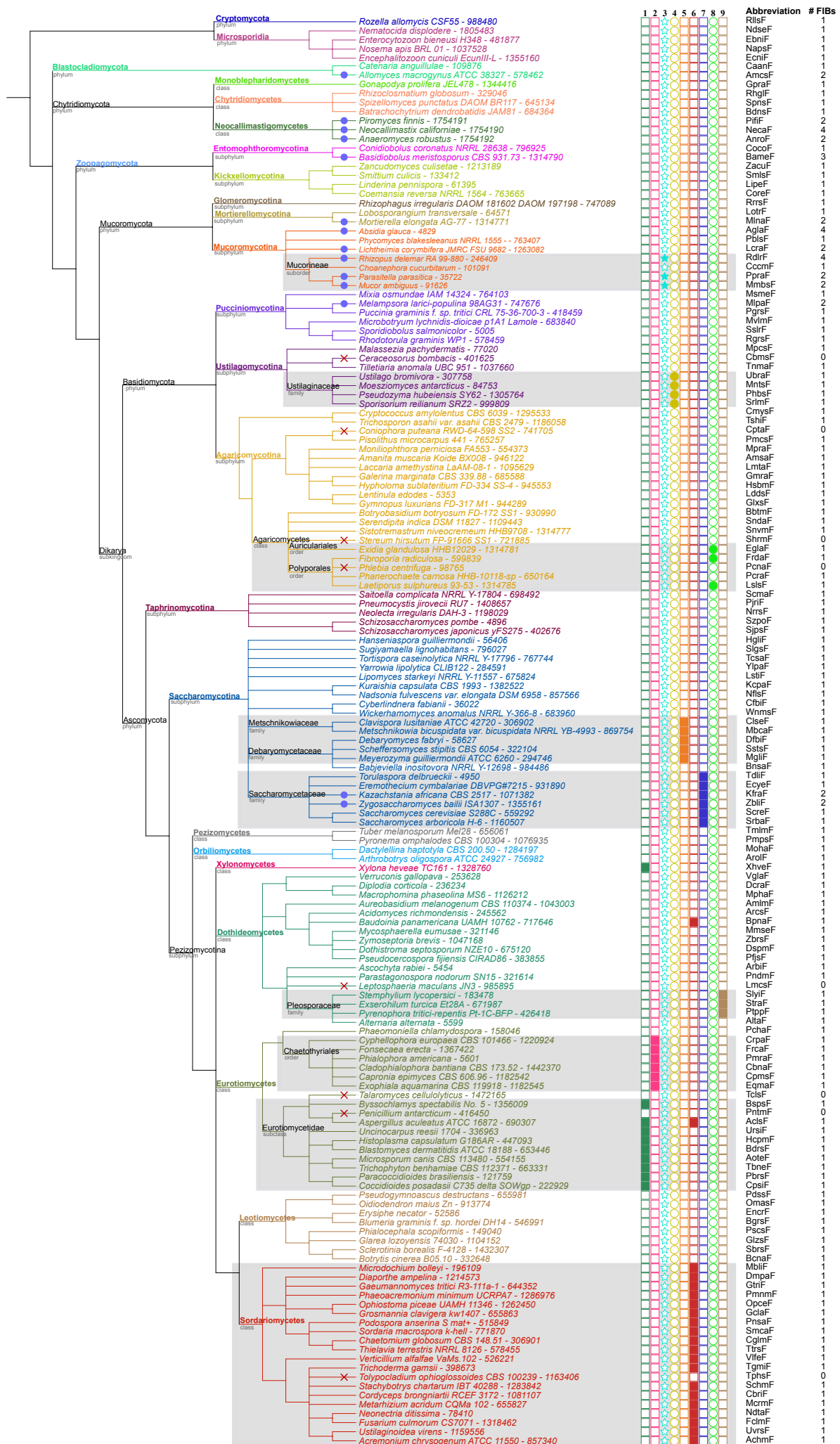

Supplement: S4 Fig — Phylogenetic relationships of the 157 fungal species for FIB proteins in the present study. Tree branches are not at scale and only depict the species relationships (topology). The species tree was initially constructed based on the NCBI taxonomy IDs (each species TaxID is indicated after a dash ‘-’ in its corresponding label name) with ETE 3 v3.1.1 [106] and visualized in ITOL 4.2.3 [107]. The tree was manually modified to fit the cladogram of the Fungi kingdom proposed by [39]. Relevant taxonomic groups in internal nodes and branches. A cross in a branch leading to a species name indicates a possible loss of the FIB protein in that species, and a circle indicates two or more FIB duplicates. To the right of the tree a presence/absence matrix (color filled figures [presence], open figure [absence]) indicating the presence of synteny communities in each species is depicted (numbered and colored as in S6 Fig. Communities belonging to Ascomycota, Basidiomycota, and Mucorinae are depicted as squares, circles, and stars, respectively. Information regarding the species abbreviations used in the present study and the number of FIB proteins is presented. (PDF) [file pcbi.1008318.s004.pdf]

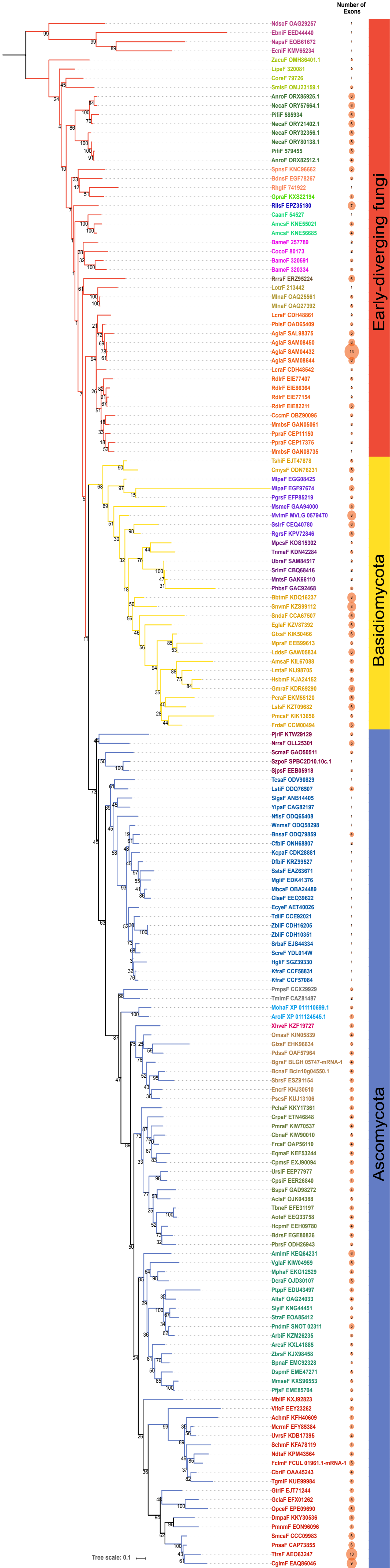

S5 Fig

Supplement: S5 Fig — The tree was rooted in the branch leading to the Microsporidia clade. The number of exons to the right of each leaf label with orange circles, whose size is proportional to the number of exons. The explicit number of exons inside each circle. The total 170 fungal FIB protein sequences were firstly aligned to a custom HMM-FIB model with hmmalign in HMMER3 3.1b2 [97], the C-terminal region outside the FIB domain was removed, and the N-terminal region (containing the GAR sequence) was independently aligned with MUSCLE v3.8.31 [101] in UGENE v1.31.0 [100]. The resultant alignment was trimmed with TrimAl v1.2rev59 with the [-automated1] option v3.8.31. The final alignment consisted of 288 sites including the FIB and GAR domains. Phylogenetic inference was performed with RaxML v8.2.12 [105] using the WAG+I+F model (best fitted for these data) and 500 bootstrap replicates, determined by the bootstopping criterion, e.g. [-autoMRE] option. (PDF) [file pcbi.1008318.s005.pdf]

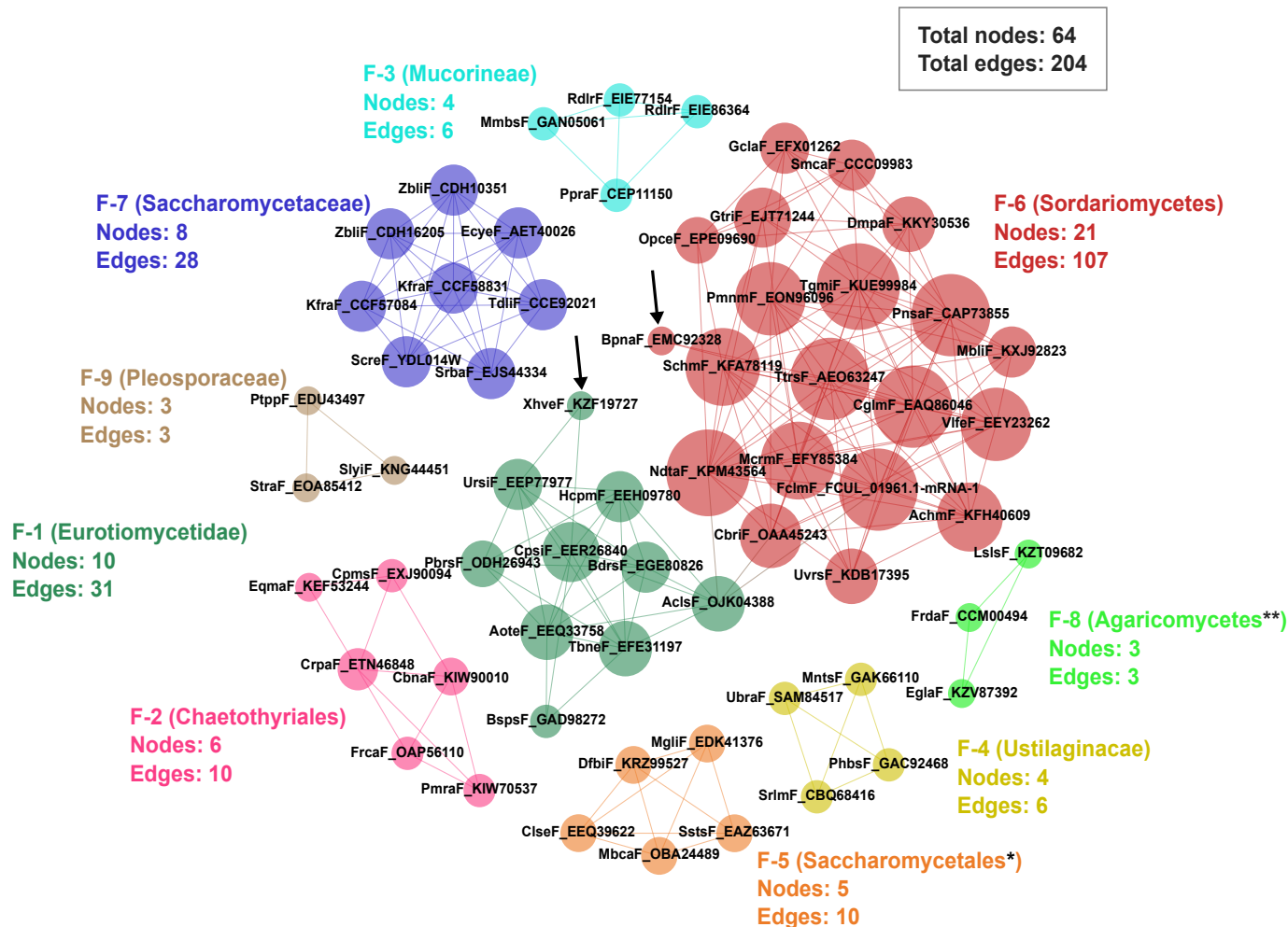

S6 Fig

Supplement: S6 Fig — Nine synteny network communities found at k-clique = 3. Nodes represent fungal FIB proteins and edges represent pairwise synteny relationships. Nodes sizes are proportional to the number of synteny connections they share. Nodes marked with black arrows indicate XhveF_KZF19727 and BpnaF_EMC92328 FIB proteins of Xylona heveae and Baudoinia panamericana, respectively. *Only comprises Metschnikowiaceae and Debaryomycetaceae families within Saccharomycetales; **Only comprises Auriculariales and Polyporales orders within Agaricomycetes (see S2 Fig). (PDF) [file pcbi.1008318.s006.pdf]

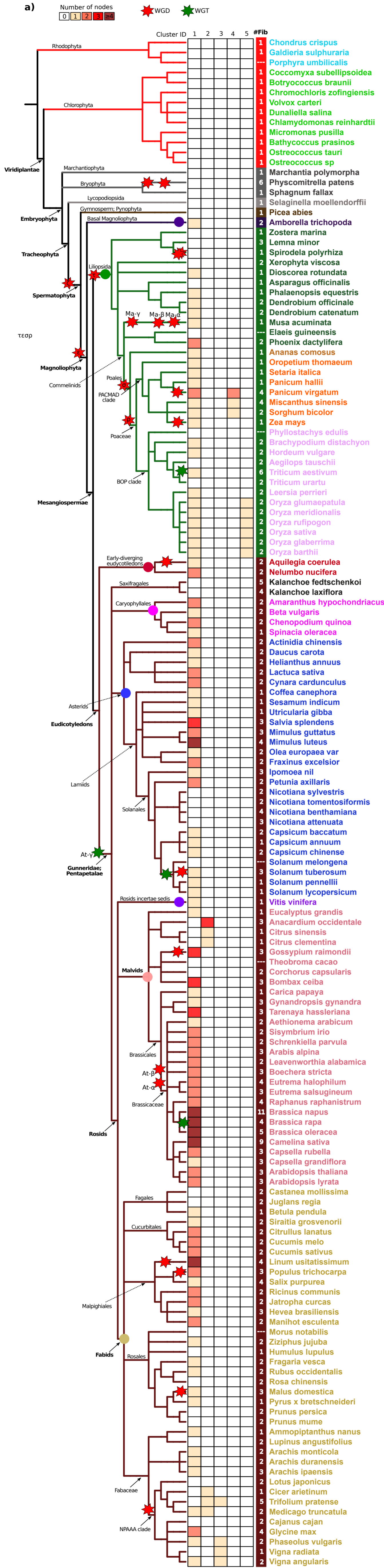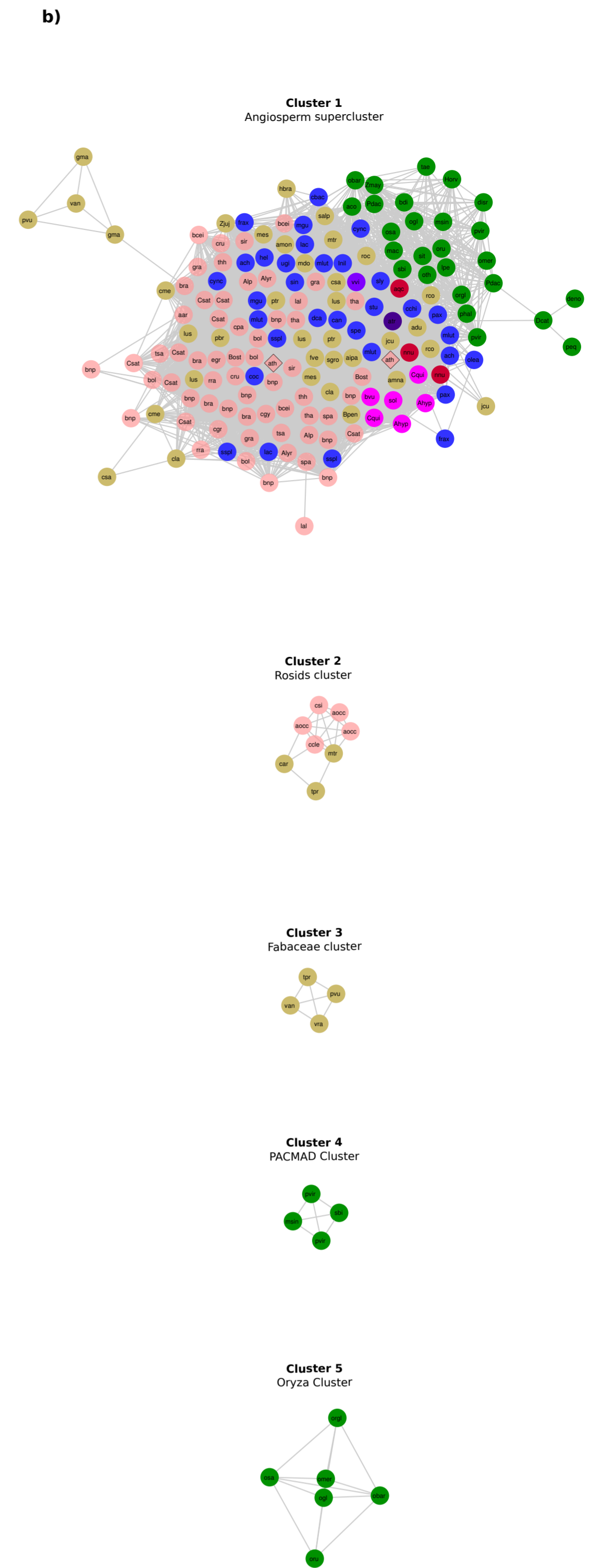

Supplement: S9 Fig — a) Phylogenetic relationships of the 328 plant species for FIB proteins in the present study. Tree branches are not at scale and only depict the species relationships (topology). The species tree was initially constructed based on the NCBI taxonomy IDs (each species TaxID is indicated after a dash ‘-’ in its corresponding label name) with ETE 3 v3.1.1 [106] and visualized in ITOL 4.2.3 [107]. To the right of the tree a presence (closed figure)/absence(open figure) matrix indicating the presence of synteny communities in each species is depicted (numbered and colored as in Fig 5). b) Microsynteny clusters of the total 223 FIB proteins from plants. Six communities were clustered according the clique = 3 to find dense communities of synteny homologous proteins. (PDF) [file pcbi.1008318.s009.pdf]

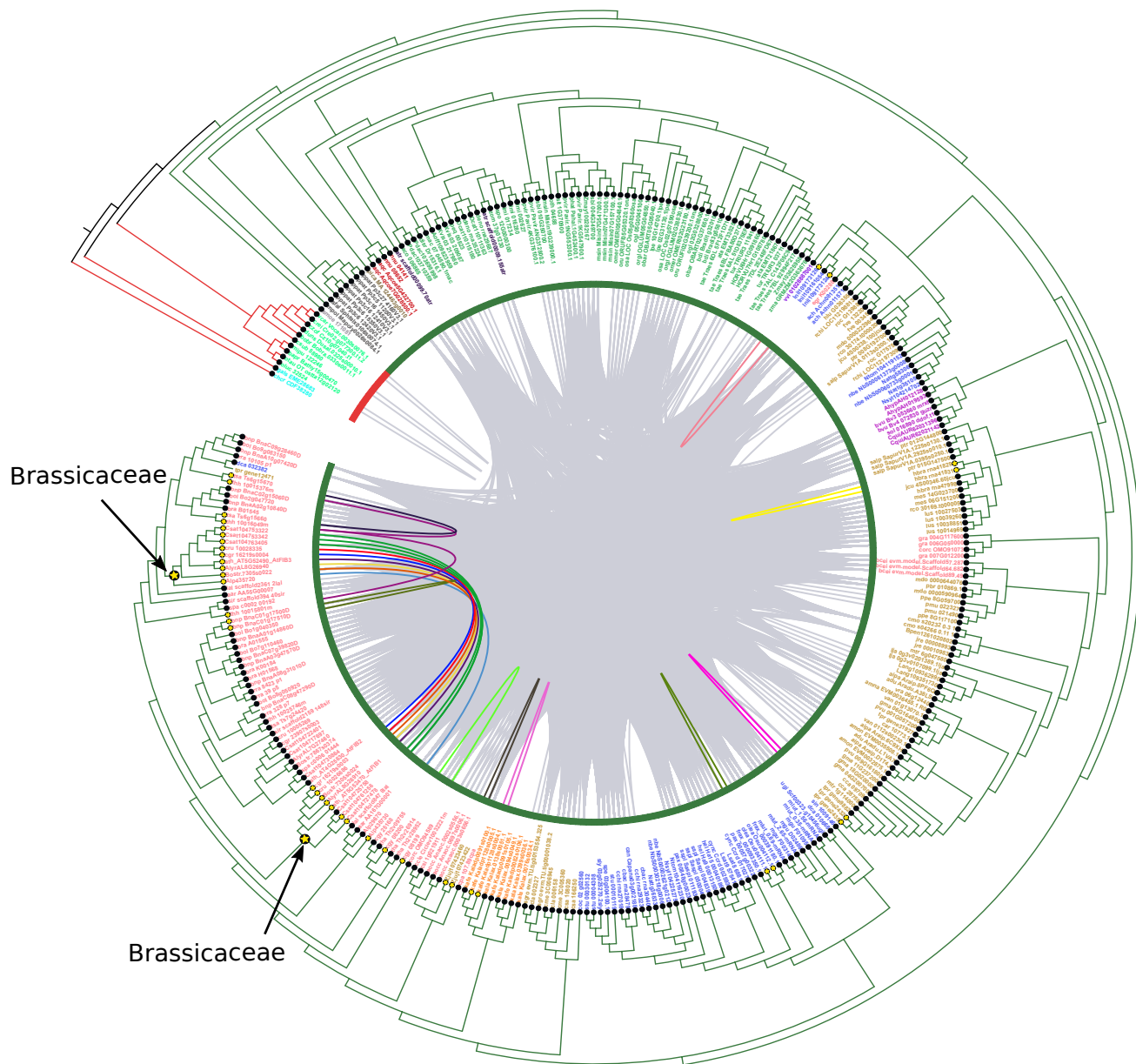

#### Color code for species IDs in the tree

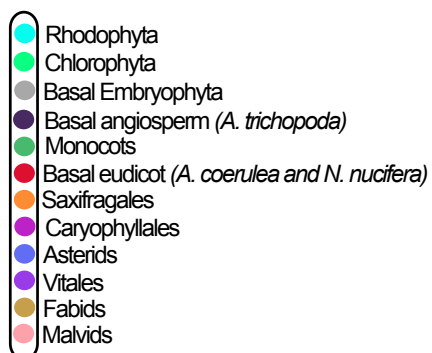

#### Color code for inner strips

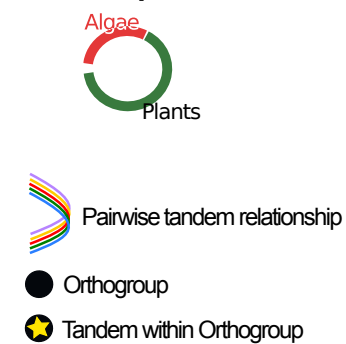

Supplement: S10 Fig — Colors of lines used only for easy visualization and has not special meaning. Grey lines connections into the tree represent the syntenic communities showed in Fig 5. The color-coded names of genes on the tree are according to their taxonomic affiliation as indicated on the right. Inner strips colored according to major taxonomic groups: algae (red) and angiosperms (green). Black filled circles on the tip of the leaves represent genes belonging to the unique orthogroup detected in plants. Yellow starts inside the black filled circles represent genes expanded by tandem duplication and yellow stars on the nodes of the tree (only two) represent clades that expanded by tandem duplication events. The total 328 plant FIB protein sequences were firstly aligned to a custom HMM-FIB model with hmmalign in HMMER3 3.1b2 [97], the C-terminal region outside the FIB domain was removed, and the N-terminal region (containing the GAR sequence) was independently aligned with MUSCLE v3.8.31 [101] in UGENE v1.31.0 [100]. Phylogenetic inference was performed with RaxML v8.2.12 [105] using the JTT+I+F model (best fitted for these data) and 500 bootstrap replicates, determined by the bootstopping criterion, e.g. [-autoMRE] option. (PDF) [file pcbi.1008318.s010.pdf]

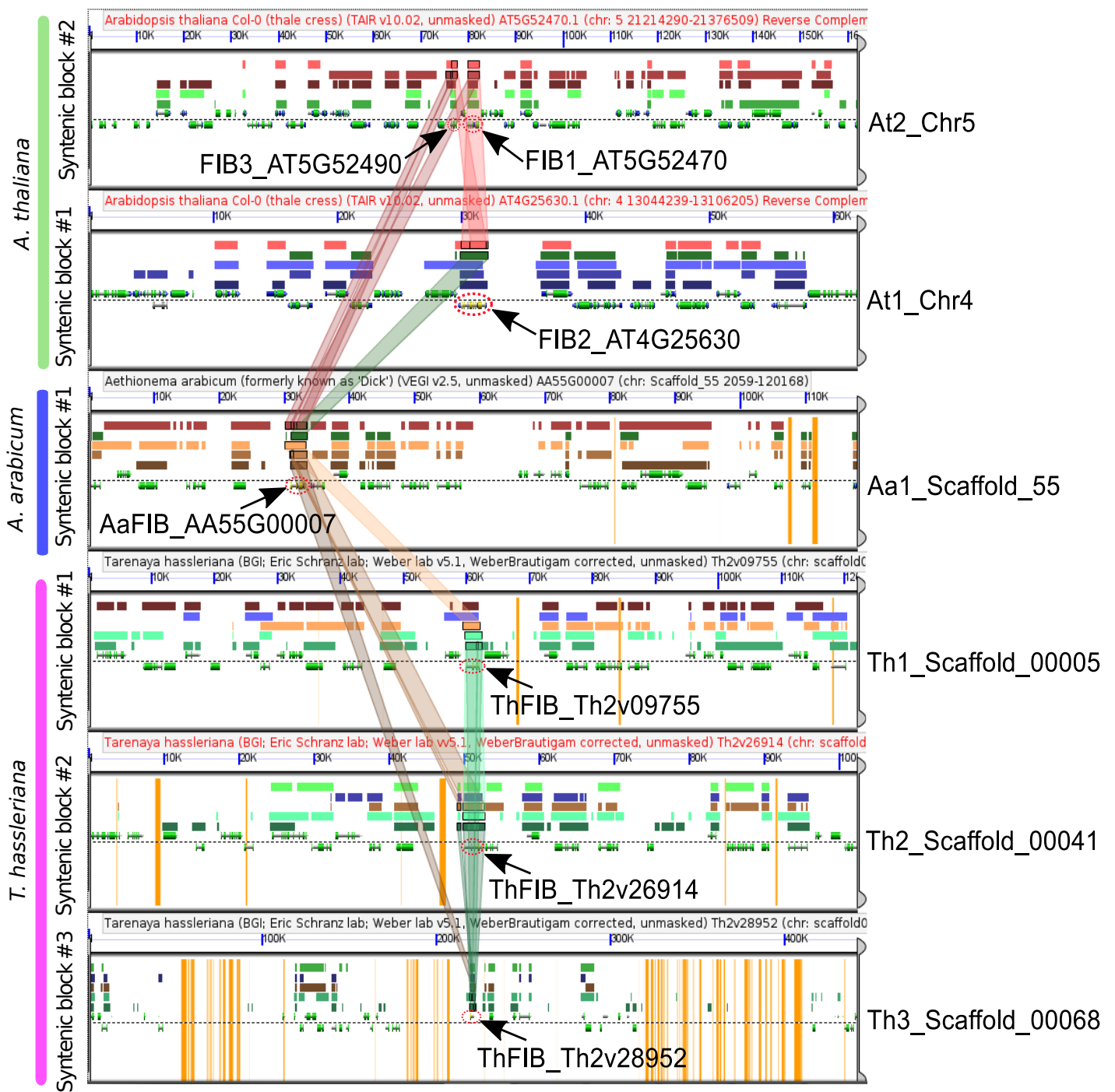

Supplement: S11 Fig — The synteny analysis show the consequence of the WGD and the different fractionation patterns in each group. A. arabicum, the early-branching of the rest of Brassicaceae, contains only one FIB protein in comparison to A. thaliana that host three FIB proteins into two duplicated blocks (one of them [AtFIB3] in Chr5 created by tandem duplication). T. hassleriana, from the Cleomaceae sister family for Brassicaceae, has undergone an independent genome triplication (Th-α), which raise three syntenic blocks in comparison to A. arabicum. Colored lines indicate syntenic relationship of FIB genes between syntenic blocks. Chr = chromosome. The analysis can be regenerated in http://genomeevolution.org/r/numm. (PDF) [file pcbi.1008318.s011.pdf]

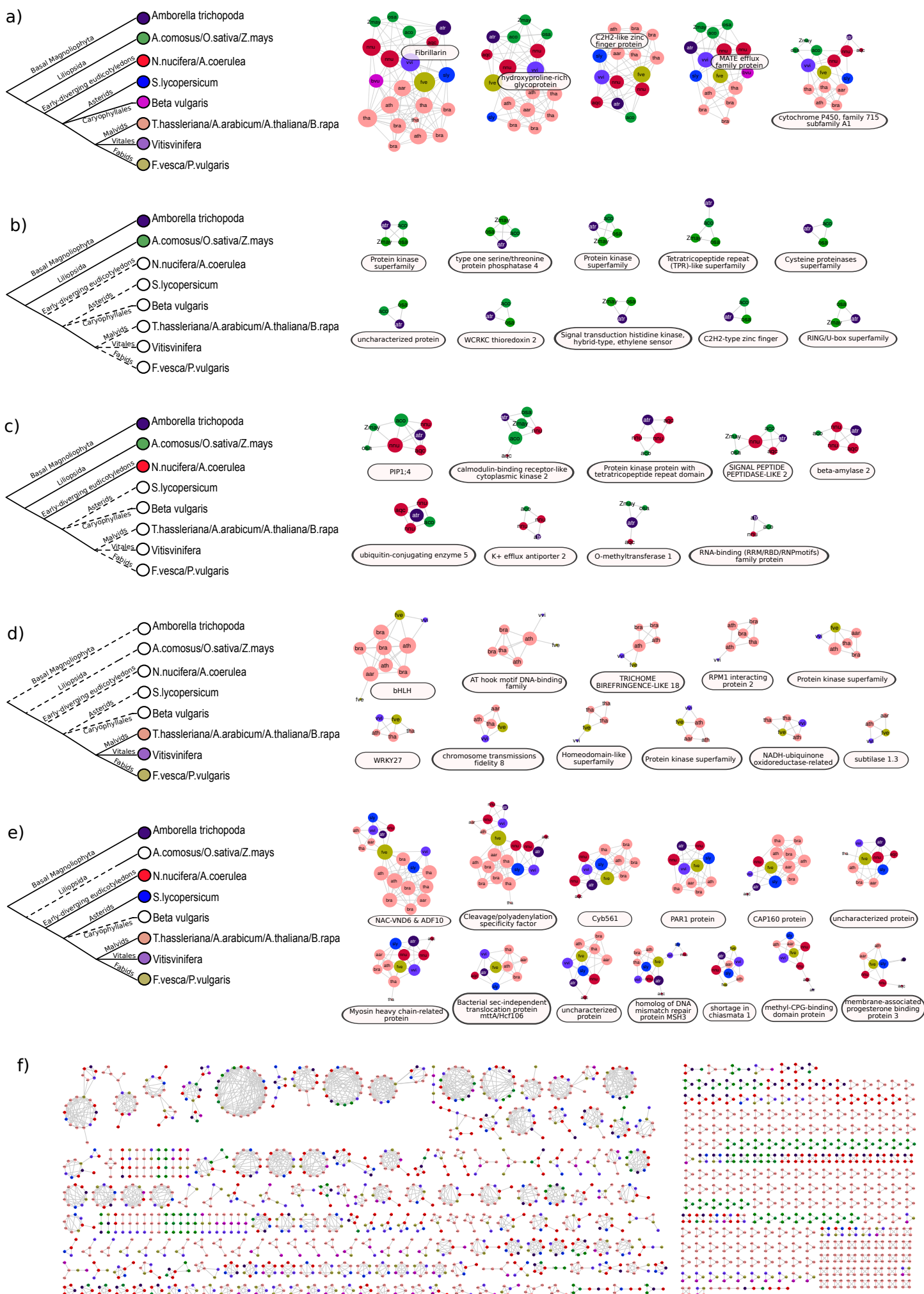

Supplement: S12 Fig — a Depiction of the microsynteny networks of the five genes that were conserved through plant linage. b) Depiction of syntenic genes shared only by amborella and monocots. These genes were not found in eudicots. c) Depiction of syntenic genes that were conserved through amborella, monocots and basal eudicots, but not the core eudicots. d) Depiction of syntenic genes that were shared only by Rosids, but not other eudicots nor monocots. These genes were probably gained in eudicot evolution. e) Depiction of syntenic genes shared by almost all angiosperms but lost in the syntenic blocks of monocots. f) Depiction of the total of genes found into the syntenic blocks of FIB genes. Communities above k-clique = 2 were not depicted due its large amount of genes in this category. (PDF) [file pcbi.1008318.s012.pdf]

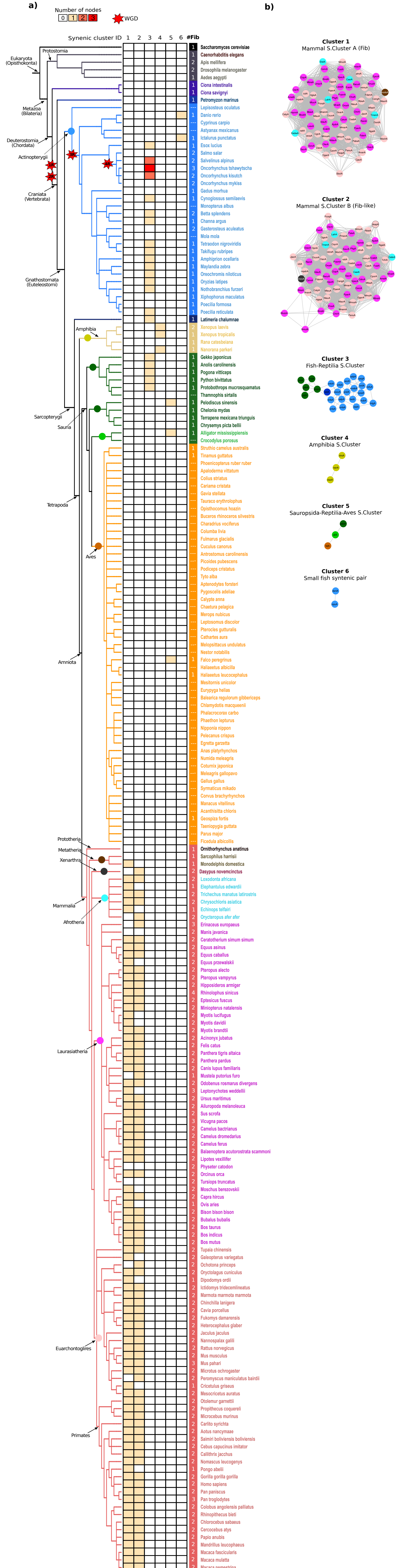

Supplement: S13 Fig — a) Phylogenetic relationships of the 195 animal species for FIB proteins in the present study. Tree branches are not at scale and only depict the species relationships (topology). The species tree was initially constructed based on the NCBI taxonomy IDs (each species TaxID is indicated after a dash ‘-’ in its corresponding label name) with ETE 3 v3.1.1 [106] and visualized in ITOL 4.2.3 [107]. To the right of the tree a presence (closed figure)/absence(open figure) matrix indicating the presence of synteny communities in each species is depicted (numbered and colored as in Fig 5). b) Microsynteny clusters of the total 197 syntenic FIB proteins from vertebrates. Six communities were clustered and coloring according Fig 6. (PDF) [file pcbi.1008318.s013.pdf]

a)

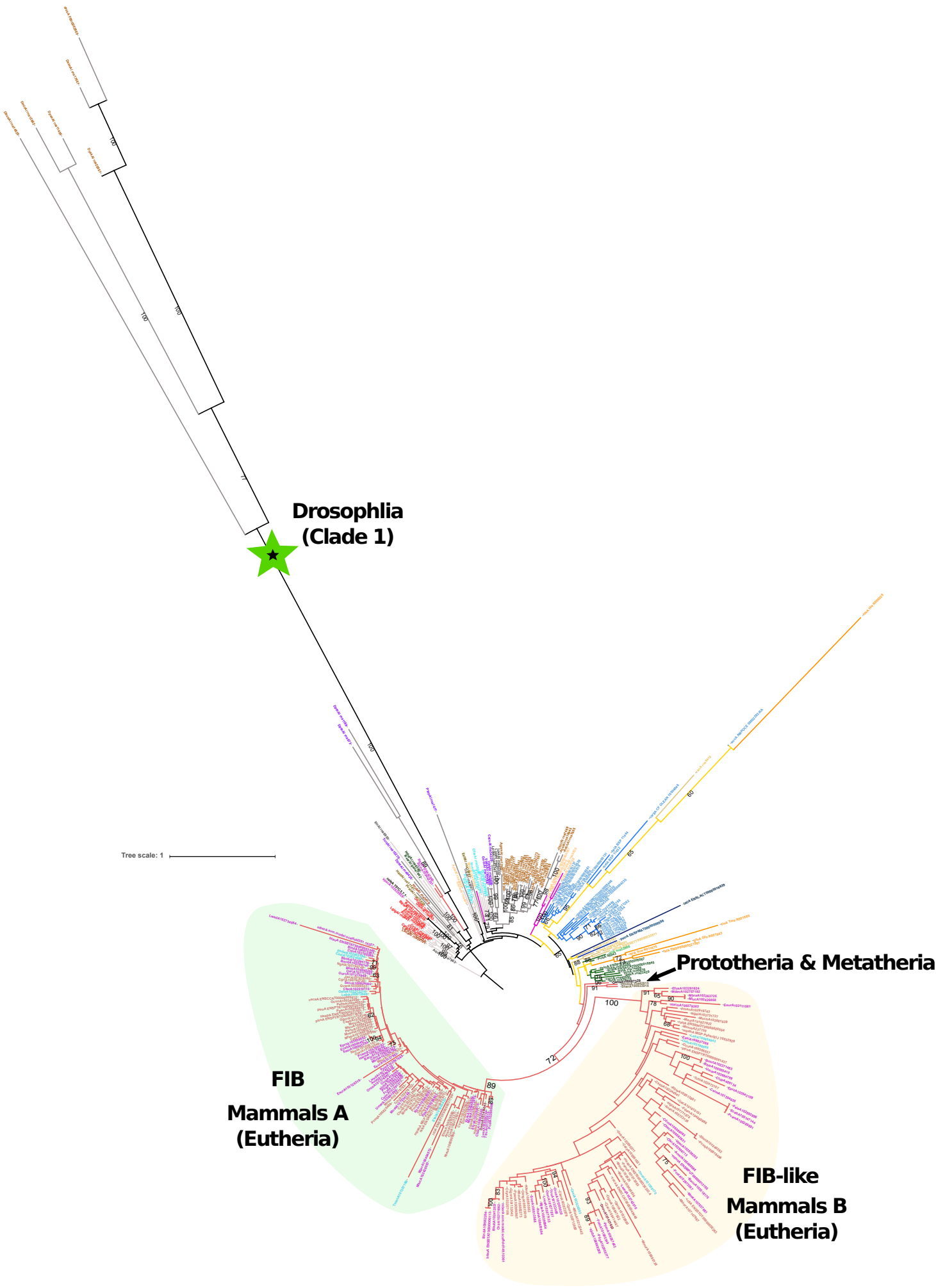

b)

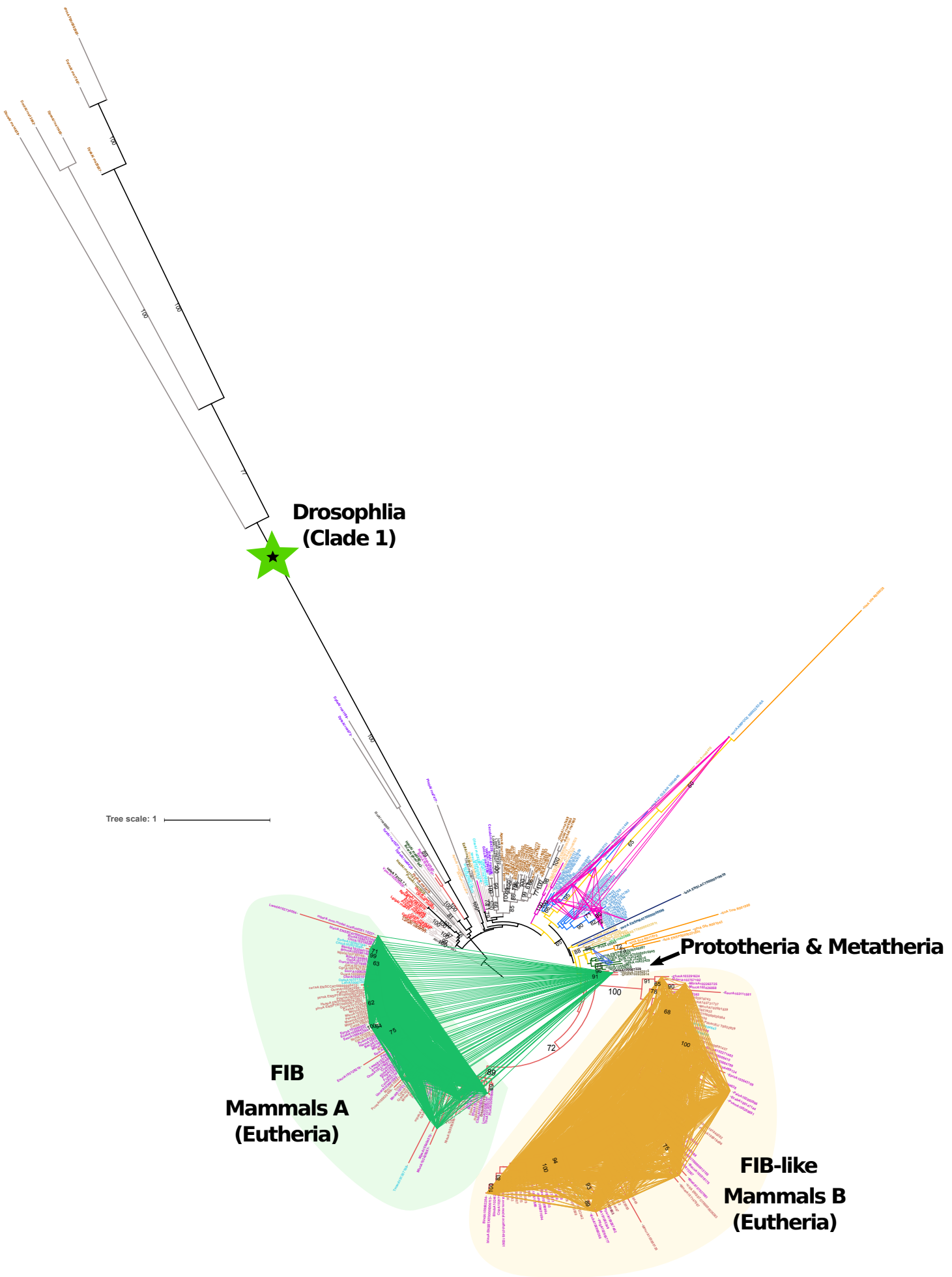

Supplement: S15 Fig — a) Phylogenetic tree with branch lengths of the total 319 detected FIB proteins shown the major clades of mammals (FIB and FIB-like). b) Phylogenetic tree with branch lengths of the total 319 detected FIB proteins and showing the syntenic connections of mammal clusters. The FIB cluster remains connected to basal Theria groups, showing evidence of ancient block conservation of this group. FIB-like cluster (yellow links) transposed to another genomic context but remains syntenic in eutheria mammals. Color-code of the names of genes on the tree are according Fig 6. (PDF) [file pcbi.1008318.s015.pdf]

## Western Blot

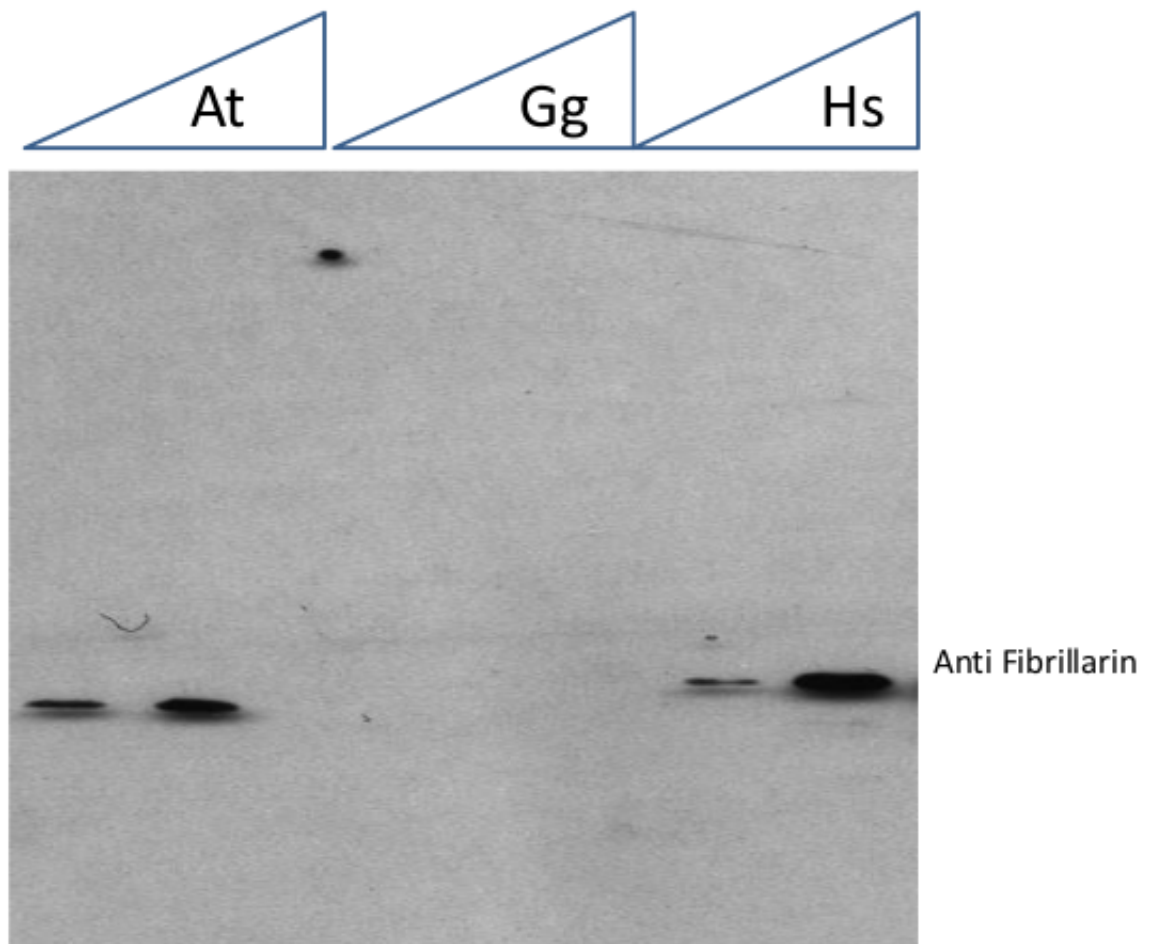

At= *Arabidopsis thaliana*  
Gg = *Gallus gallus*  
Hs = *Homo sapiens*

Supplement: S16 Fig — We decided to test if birds lack FIBs as the genomic data suggest. We carried out extracts from the whole Arabidopsis thaliana plant, heart tissue from Gallus gallus domesticus, as a representatives of the avian clade, and extract from human cells (HeLa cells), as a representative of Mammals. We used the commercial antibody from abcam ab166630. (PDF) [file pcbi.1008318.s016.pdf]
