# Supplementary material for: Fibrillarin evolution through the Tree of Life: Comparative genomics and microsynteny network analyses provide new insights into the evolutionary history of Fibrillarin
Source: PLoS Comput Biol. 2020 Oct 19;16(10):e1008318. doi: 10.1371/journal.pcbi.1008318 (PMC7608942; doi:10.1371/journal.pcbi.1008318)
Supplement: S8 Fig — Network representation of the protein-coding genes contained within the same synteny block indexes as fungal FIB homologues. Nodes represent proteins and edges represent pairwise synteny relationships. Node sizes are proportional to the number of synteny connections per node (degree), however these sizes are not comparable among independent networks. To construct these networks, we retrieved all pairwise relationships (edges) between proteins (nodes) that matched the same block indexes (indicated in S11 Table) as the fungal FIB homologues found within each of the nine fungal FIB communities (S6 Fig). Then, we used CFinder at k-clique = 3 to find communities of synteny homologous proteins; the original fungal FIB communities were also recovered (S6 Fig). For easier visualization, communities with low number of nodes were filtered out and we only depict communities with a determined number of nodes or above (indicated by ‘AF’ in the figure; the applied filter was arbitrarily chosen for each network). Colors were set to help define each community. The complete sets of nodes and edges, before and after CFinder analysis are listed in S11 and S12 Tables, respectively. Taking into the account the number of syntelogs but not the number of species, the biggest syntenic block corresponded to the F-4 FIB community, which was composed of at least 40 syntelogs from the Ustilaginaceae family (S8D Fig). The smallest syntenic blocks, taking into account both the number of syntelogs and the number species, were F-3, F-8, and F-9 (S8C, S8H and S8I Fig). F-1, F-6, and F-7 were the biggest syntenic blocks when number of syntelogs and number of species were taken into account (S8A, S8F and S8G Fig). Abbreviations used in the networks: Original FIB community (FC); total number of communities at k-clique = 3 (TC); range of number of nodes per community at k-clique = 3 (RN); number of communities depicted (CD); and applied filter (AF, the minimum number of nodes per community). a) F-1 (Eur [file pcbi.1008318.s008.pdf]

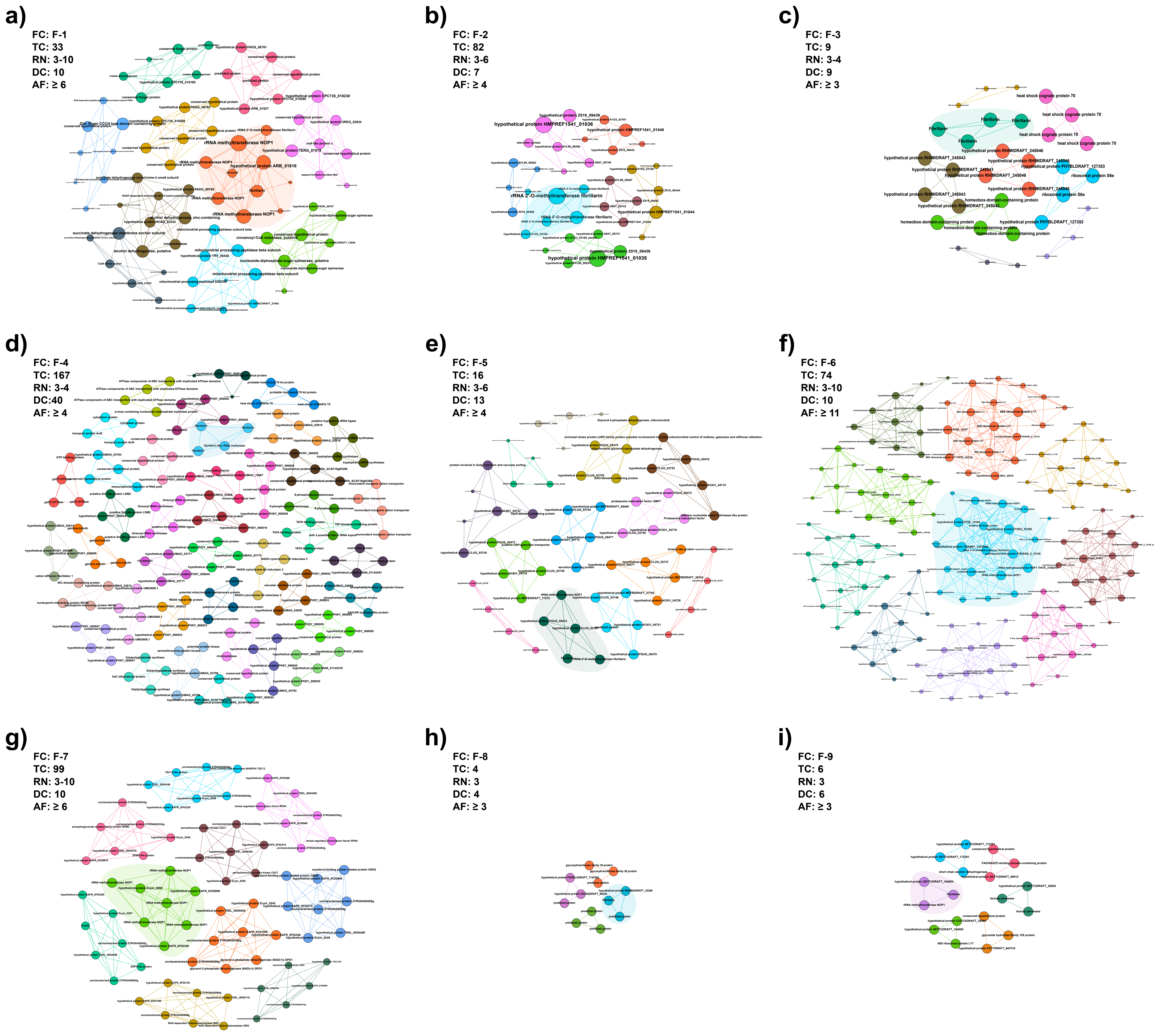
a)
FC: F-1
TC: 33
RN: 3-10
DC: 10
AF: ≥ 6
b)
FC: F-2
TC: 82
RN: 3-6
DC: 7
AF: ≥ 4
c)
FC: F-3
TC: 9
RN: 3-4
DC: 9
AF: ≥ 3
d)
FC: F-4
TC: 167
RN: 3-4
DC:40
AF: ≥ 4
e)
FC: F-5
TC: 16
RN: 3-6
DC: 13
AF: ≥ 4
f)
FC: F-6
TC: 74
RN: 3-10
DC: 10
AF: ≥ 11
g)
FC: F-7
TC: 99
RN: 3-10
DC: 10
AF: ≥ 6
h)
FC: F-8
TC: 4
RN: 3
DC: 4
AF: ≥ 3
i)
FC: F-9
TC: 6
RN: 3
DC: 6
AF: ≥ 3

S8 Fig
