# Supplementary material for: Fibrillarin evolution through the Tree of Life: Comparative genomics and microsynteny network analyses provide new insights into the evolutionary history of Fibrillarin
Source: PLoS Comput Biol. 2020 Oct 19;16(10):e1008318. doi: 10.1371/journal.pcbi.1008318 (PMC7608942; doi:10.1371/journal.pcbi.1008318)
Supplement: S14 Fig — a) Depiction of microsynteny networks of genes into the “FIB microsynteny block”, that were conserved through mammalian species, but not the eutherian D. novemcinctus. Only microsynteny networks containing the metatherian M. domestica are shown. b) Depiction of microsynteny networks genes into the “FIB-like microsynteny block”, that were conserved through eutherian mammals. Only microsynteny networks containing the Xenarthra D. novemcinctus are shown. FIB and FIB-like synteny blocks remains in different genomic contexts throughout mammalian evolution. (PDF) [file pcbi.1008318.s014.pdf]

a)

Neighbouring genes at the FIB syntenic block

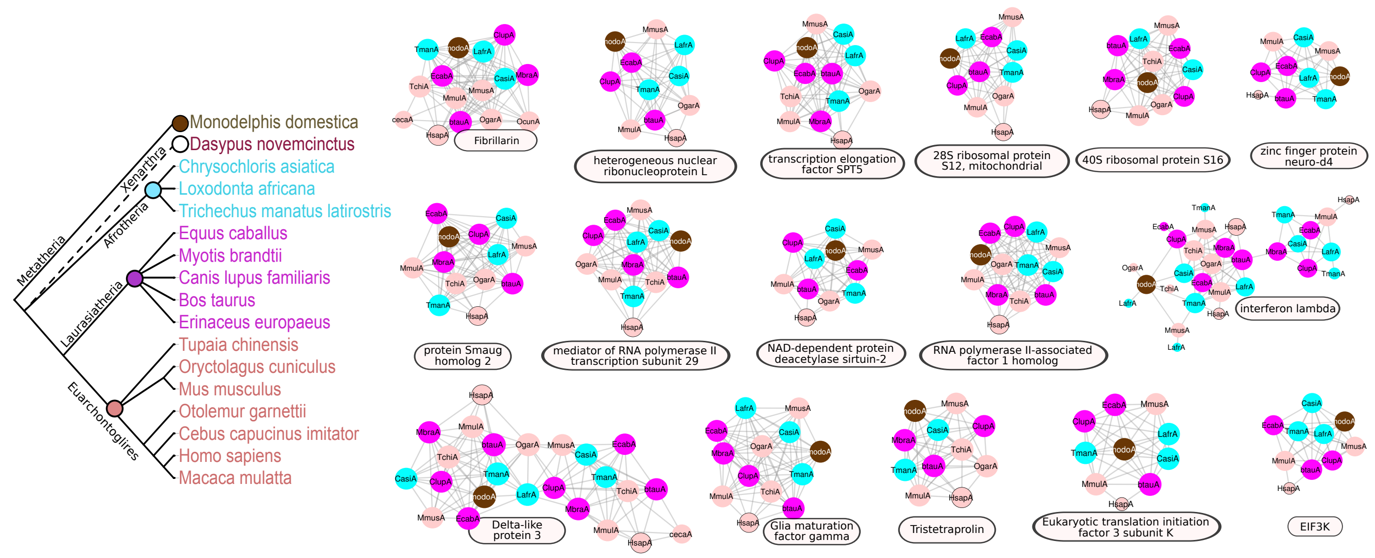

b)

Neighbouring genes at the FIB-like syntenic block

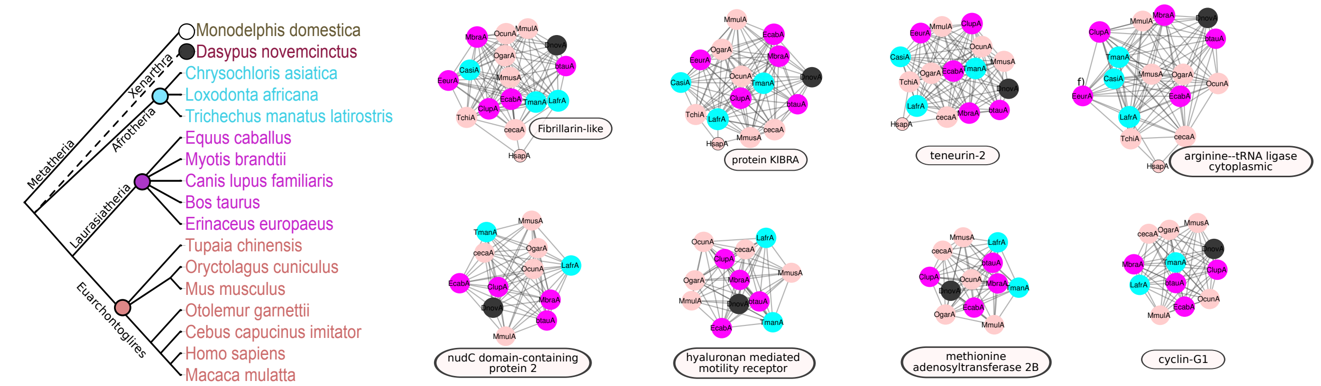

S14 Fig
