# Supplementary material for: Fibrillarin evolution through the Tree of Life: Comparative genomics and microsynteny network analyses provide new insights into the evolutionary history of Fibrillarin
Source: PLoS Comput Biol. 2020 Oct 19;16(10):e1008318. doi: 10.1371/journal.pcbi.1008318 (PMC7608942; doi:10.1371/journal.pcbi.1008318)
Supplement: S7 Fig — a) Phylogenetic relationships of the 157 fungal species for FIB proteins in the present study. Tree branches are not at scale and only depict the species relationships (topology). The species tree was initially constructed based on the NCBI taxonomy IDs (each species TaxID is indicated after a dash ‘-’ in its corresponding label name) with ETE 3 v3.1.1 [106] and visualized in ITOL 4.2.3 [107]. The tree was manually modified to fit the cladogram of the Fungi kingdom proposed by [39]. Relevant taxonomic groups in internal nodes and branches. A cross in a branch leading to a species name indicates a possible loss of the FIB protein in that species, and a circle indicates two or more FIB duplicates. To the right of the tree a presence (closed figure)-absence(open figure) matrix indicating the presence of synteny communities in each species is depicted (numbered and colored as in S6 Fig). Communities belonging to Ascomycota, Basidiomycota, and Mucorinae depicted as squares, circles, and stars, respectively. Information regarding the species abbreviations used in the present study and the number of FIB proteins also presented. b) Microsynteny clusters in fungi. Eight communities were clustered and colored according S6 Fig. (PDF) [file pcbi.1008318.s007.pdf]

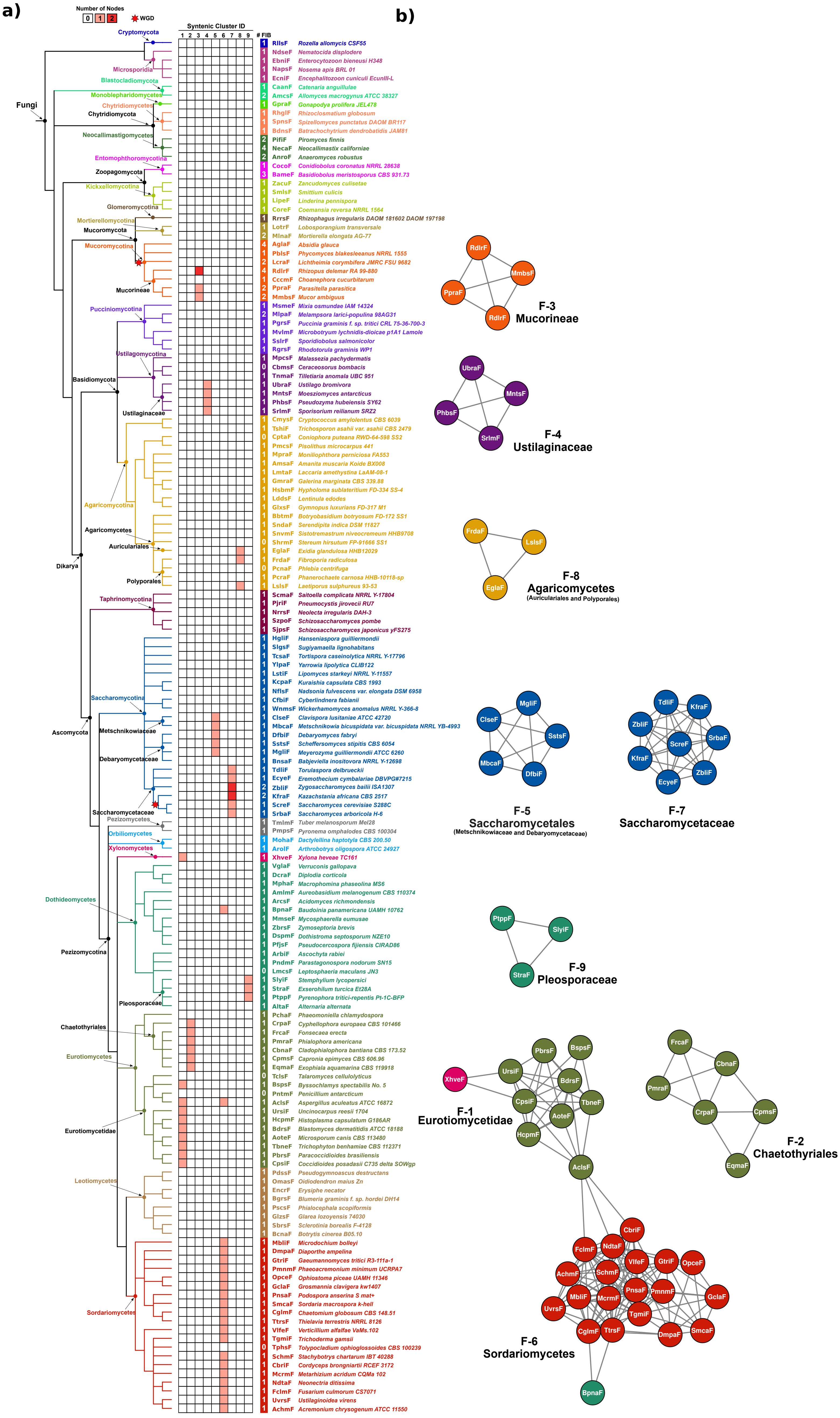

a)
Number of Nodes
0 1 2
WGD
Syntenic Cluster ID
1 2 3 4 5 6 7 8 9
FIB
b)
Fungi
Cryptomycota
Microsporidia
Blastocladiomycota
Monoblepharidomycetes
Chytridiomycetes
Chytridiomycota
Neocallimastigomycetes
Entomophthoromycotina
Zoopagomycota
Kickxellomycotina
Glomeromycotina
Mortierellomycotina
Mucoromycota
Mucoromycotina
Mucorineae
Pucciniomycotina
Ustilagomycotina
Basidiomycota
Ustilaginaceae
Agaricomycotina
Agaricomycetes
Auriculariales
Dikarya
Polyporales
Taphrinomycotina
Saccharomycotina
Ascomycota
Metschnikowiaceae
Debaryomycetaceae
Saccharomycetaceae
Pezizomycetes
Orbiliomycetes
Xylonomycetes
Dothideomycetes
Pezizomycotina
Pleosporaceae
Chaetothyriales
Eurotiomycetes
Eurotiomycetidae
Leotiomycetes
Sordariomycetes
1 RllsF Rozella allomycis CSF55
1 NdseF Nematocida displodere
1 EbniF Enterocytozoon bieneusi H348
1 NapsF Nosema apis BRL 01
1 EcniF Encephalitozoon cuniculi EcunIII-L
1 CaanF Catenaria anguillulae
2 AmcsF Allomyces macrogynus ATCC 38327
1 GpraF Gonapodya prolifera JEL478
1 RhglF Rhizoclosmatium globosum
1 SpnsF Spizellomyces punctatus DAOM BR117
1 BdnsF Batrachochytrium dendrobatidis JAM81
2 PifiF Piromyces finnis
4 NecaF Neocallimastix californiae
2 AnroF Anaeromyces robustus
1 CocoF Conidiobolus coronatus NRRL 28638
3 BameF Basidiobolus meristosporus CBS 931.73
1 ZacuF Zancudomyces culisetae
1 SmlsF Smittium culicis
1 LipeF Linderina pennispora
1 CoreF Coemansia reversa NRRL 1564
1 RrrsF Rhizophagus irregularis DAOM 181602 DAOM 197198
1 LotrF Lobosporangium transversale
2 MlnaF Mortierella elongata AG-77
4 AglaF Absidia glauca
1 PblsF Phycomyces blakesleeanus NRRL 1555
2 LcraF Lichtheimia corymbifera JMRC FSU 9682
4 RdlrF Rhizopus delemar RA 99-880
1 CccmF Choanephora cucurbitarum
2 PpraF Parasitella parasitica
2 MmbsF Mucor ambiguus
1 MsmeF Mixia osmundae IAM 14324
2 MlpaF Melampsora larici-populina 98AG31
1 PgrsF Puccinia graminis f. sp. tritici CRL 75-36-700-3
1 MvlmF Microbotryum lychnidis-dioicae p1A1 Lamole
1 SslrF Sporidiobolus salmonicolor
1 RgrsF Rhodotorula graminis WP1
1 MpcsF Malassezia pachydermatis
0 CbmsF Ceraceosorus bombacis
1 TnmaF Tilletiaria anomala UBC 951
1 UbraF Ustilago bromivora
1 MntsF Moesziomyces antarcticus
1 PhbsF Pseudozyma hubeiensis SY62
1 SrlmF Sporisorium reilianum SRZ2
1 CmysF Cryptococcus amylolentus CBS 6039
1 TshiF Trichosporon asahii var. asahii CBS 2479
0 CptaF Coniophora puteana RWD-64-598 SS2
1 PmcsF Pisolithus microcarpus 441
1 MpraF Moniliophthora perniciosa FA553
1 AmsaF Amanita muscaria Koide BX008
1 LmtaF Laccaria amethystina LaAM-08-1
1 GmraF Galerina marginata CBS 339.88
1 HsbmF Hypholoma sublateritium FD-334 SS-4
1 LddsF Lentinula edodes
1 GlxsF Gymnopus luxurians FD-317 M1
1 BbtmF Botryobasidium botryosum FD-172 SS1
1 SndaF Serendipita indica DSM 11827
1 SnvmF Sistotremastrum niveocremeum HHB9708
0 ShrmF Stereum hirsutum FP-91666 SS1
1 EglaF Exidia glandulosa HHB12029
1 FrdaF Fibroporia radiculosa
0 PcnaF Phlebia centrifuga
1 PcraF Phanerochaete carnosa HHB-10118-sp
1 LslsF Laetiporus sulphureus 93-53
1 ScmaF Saitoella complicata NRRL Y-17804
1 PjriF Pneumocystis jirovecii RU7
1 NrrsF Neolecta irregularis DAH-3
1 SzpoF Schizosaccharomyces pombe
1 SjpsF Schizosaccharomyces japonicus yFS275
1 HgliF Hanseniaspora guilliermondii
1 SlgsF Sugiyamaella lignohabitans
1 TcsaF Tortispora caseinolytica NRRL Y-17796
1 YlpaF Yarrowia lipolytica CLIB122
1 LstiF Lipomyces starkeyi NRRL Y-11557
1 KcpaF Kuraishia capsulata CBS 1993
1 NflsF Nadsonia fulvescens var. elongata DSM 6958
1 CfbiF Cyberlindnera fabianii
1 WnmsF Wickerhamomyces anomalus NRRL Y-366-8
1 ClseF Clavispora lusitaniae ATCC 42720
1 MbcaF Metschnikowia bicuspidata var. bicuspidata NRRL YB-4993
1 DfbiF Debaryomyces fabryi
1 SstsF Scheffersomyces stipitis CBS 6054
1 MgliF Meyerozyma guilliermondii ATCC 6260
1 BnsaF Babjeviella inositovora NRRL Y-12698
1 TdliF Torulaspora delbrueckii
1 EcyeF Eremothecium cymbalariae DBVPG#7215
2 ZbliF Zygosaccharomyces bailii ISA1307
2 KfraF Kazachstania africana CBS 2517
1 ScreF Saccharomyces cerevisiae S288C
1 SrbaF Saccharomyces arboricola H-6
1 TmlmF Tuber melanosporum Mel28
1 PmpsF Pyronema omphalodes CBS 100304
1 MohaF Dactylellina haptotyla CBS 200.50
1 AroIF Arthrobotrys oligospora ATCC 24927
1 XhveF Xylona heveae TC161
1 VglaF Verruconis gallopava
1 DcraF Diplodia corticola
1 MphaF Macrophomina phaseolina MS6
1 AmlmF Aureobasidium melanogenum CBS 110374
1 ArcsF Acidomyces richmondensis
1 BpnaF Baudoinia panamericana UAMH 10762
1 MmseF Mycosphaerella eumusae
1 ZbrsF Zymoseptoria brevis
1 DspmF Dothistroma septosporum NZE10
1 PfjsF Pseudocercospora fijiensis CIRAD86
1 ArbiF Ascochyta rabiei
1 PndmF Parastagonospora nodorum SN15
0 LmcsF Leptosphaeria maculans JN3
1 SlyiF Stemphylium lycopersici
1 StraF Exserohilum turcica Et28A
1 PtppF Pyrenophora tritici-repentis Pt-1C-BFP
1 AltaF Alternaria alternata
1 PchaF Phaeomoniella chlamydospora
1 CrpaF Cyphellophora europaea CBS 101466
1 FrcaF Fonsecaea erecta
1 PmraF Phialophora americana
1 CbnaF Cladophialophora bantiana CBS 173.52
1 CpmsF Capronia epimyces CBS 606.96
1 EqmaF Exophiala aquamarina CBS 119918
0 TclsF Talaromyces cellulolyticus
1 BspsF Byssochlamys spectabilis No. 5
0 PntmF Penicillium antarcticum
1 AclsF Aspergillus aculeatus ATCC 16872
1 UrsiF Uncinocarpus reesii 1704
1 HcpmF Histoplasma capsulatum G186AR
1 BdrsF Blastomyces dermatitidis ATCC 18188
1 AoteF Microsporum canis CBS 113480
1 TbneF Trichophyton benhamiae CBS 112371
1 PbrsF Paracoccidioides brasiliensis
1 CpsiF Coccidioides posadasii C735 delta SOWgp
1 PdssF Pseudogymnoascus destructans
1 OmasF Oidiodendron maius Zn
1 EncrF Erysiphe necator
1 BgrsF Blumeria graminis f. sp. hordei DH14
1 PscsF Phialocephala scopiformis
1 GlzsF Glarea lozoyensis 74030
1 SbrsF Sclerotinia borealis F-4128
1 BcnaF Botrytis cinerea B05.10
1 MbliF Microdochium bolleyi
1 DmpaF Diaporthe ampelina
1 GtriF Gaeumannomyces tritici R3-111a-1
1 PmnmF Phaeoacremonium minimum UCRPA7
1 OpceF Ophiostoma piceae UAMH 11346
1 GclaF Grosmannia clavigera kw1407
1 PnsaF Podospora anserina S mat+
1 SmcaF Sordaria macrospora k-hell
1 CglmF Chaetomium globosum CBS 148.51
1 TtrsF Thielavia terrestris NRRL 8126
1 VlfeF Verticillium alfalfae VaMs.102
1 TgmiF Trichoderma gamsii
0 TphsF Tolypocladium ophioglossoides CBS 100239
1 SchmF Stachybotrys chartarum IBT 40288
1 CbriF Cordyceps brongniartii RCEF 3172
1 McrmF Metarhizium acridum CQMa 102
1 NdtaF Neonectria ditissima
1 FclmF Fusarium culmorum CS7071
1 UvrsF Ustilaginoidea virens
1 AchmF Acremonium chrysogenum ATCC 11550
RdlrF MmbsF PpraF RdlrF
F-3 Mucorineae
UbraF MntsF PhbsF SrlmF
F-4 Ustilaginaceae
FrdaF LslsF EglaF
F-8 Agaricomycetes (Auriculariales and Polyporales)
MgliF ClseF SstsF MbcaF DfbiF
F-5 Saccharomycetales (Metschnikowiaceae and Debaryomycetaceae)
TdliF KfraF ZbliF SrbaF ScreF KfraF ZbliF EcyeF
F-7 Saccharomycetaceae
PtppF SlyiF StraF
F-9 Pleosporaceae
XhveF UrsiF PbrsF BspsF BdrsF CpsiF TbneF AoteF HcpmF AclsF
F-1 Eurotiomycetidae
FrcaF CbnaF PmraF CrpaF CpmsF EqmaF
F-2 Chaetothyriales
CbriF FclmF NdtaF VlfeF GtriF OpceF AchmF SchmF PnsaF PmnmF MbliF McrmF GclaF UvrsF TgmiF CglmF TtrsF DmpaF SmcaF BpnaF
F-6 Sordariomycetes

S7 Fig
